# Conservative Management of Patent Ductus Arteriosus in Preterm Infants—A Systematic Review and Meta-Analyses Assessing Differences in Outcome Measures Between Randomized Controlled Trials and Cohort Studies

**DOI:** 10.3389/fped.2021.626261

**Published:** 2021-02-25

**Authors:** Tim Hundscheid, Esther J. S. Jansen, Wes Onland, Elisabeth M. W. Kooi, Peter Andriessen, Willem P. de Boode

**Affiliations:** ^1^Division of Neonatology, Department of Paediatrics, Radboud Institute for Health Sciences, Amalia Children's Hospital, Radboud University Medical Centre, Nijmegen, Netherlands; ^2^Department of Neonatology, Amsterdam University Medical Centers, VU University Medical Center, Emma Children's Hospital, University of Amsterdam, Amsterdam, Netherlands; ^3^Division of Neonatology, University Medical Center Groningen, Beatrix Children's Hospital, University of Groningen, Groningen, Netherlands; ^4^Department of Neonatology, Máxima Medical Center Veldhoven, Eindhoven, Netherlands; ^5^Department of Applied Physics, School of Medical Physics and Engineering, Eindhoven University of Technology, Eindhoven, Netherlands

**Keywords:** PDA, ibuprofen, indomethacin, paracetamol, ligation, placebo, morbidity, mortality

## Abstract

**Objective:** This study aims to evaluate outcome after conservative management (no pharmacological/surgical intervention other than fluid restriction, diuretics, or ventilator adjustments) compared with active (pharmacological and/or surgical) treatment for patent ductus arteriosus (PDA) in preterm infants and analyze differences in outcome between randomized controlled trials (RCTs) and cohort studies.

**Study Design:** This is a systematic literature review using PubMed, EMBASE, and Cochrane library. RCTs and cohort studies comparing conservative management with active treatment were included. Meta-analysis was used to compare conservative management with any active (pharmacological and/or surgical), any pharmacological (non-prophylactic and prophylactic), and/or surgical treatment for mortality as primary and major neonatal morbidity as secondary outcome measure. Fixed-effect analysis was used, unless heterogeneity (*I*^2^) was >50%. Outcome is presented as relative risk (RR) with 95% confidence interval.

**Results:** Twelve cohort studies and four RCTs were included, encompassing 41,804 and 720 patients, respectively. In cohort studies, conservative management for PDA was associated with a significantly higher risk for mortality (RR, 1.34 [1.12–1.62]) but a significantly lower risk for bronchopulmonary dysplasia (RR, 0.55 [0.46–0.65]), necrotizing enterocolitis (RR, 0.85 [0.77–0.93]), intraventricular hemorrhage (RR, 0.88 [0.83–0.95]), and retinopathy of prematurity (RR, 0.47 [0.28–0.79]) compared with any active PDA treatment. Meta-analysis of the RCTs revealed no significant differences in outcome between conservative management and active treatment.

**Conclusion:** No differences in mortality or morbidity for conservative management compared with active treatment regimens were observed in RCTs. Findings from cohort studies mainly highlight the lack of high-quality evidence for conservative management for PDA in preterm infants.

## Introduction

A patent ductus arteriosus (PDA) is very common in very low birth weight (VLBW) infants (<1,500 g) (1). It is associated with mortality and severe morbidity, such as bronchopulmonary dysplasia (BPD), necrotizing enterocolitis (NEC), intraventricular hemorrhage (IVH), and retinopathy of prematurity (ROP) (2).

Prophylactic treatment with indomethacin has been shown to reduce the incidence of symptomatic PDA, ligation, and severe IVH (3). Network meta-analysis of randomized controlled trials (RCTs) showed a significant effect on ductus arteriosus (DA) closure in pharmacologically treated children compared with placebo/no treatment (4). However, effective closure of the DA has not resulted in an improvement either in survival or major neonatal morbidity. When analyzing these RCTs, one has to be aware of the great heterogeneity in used definitions (and therefore inclusion criteria) for hemodynamically significant PDA (hsPDA) (5) and the remarkably high rate of open-label-treated patients in the control group (6). Ligation is an effective strategy to close the DA as well but has been associated with adverse outcome (7). Recent studies that adjusted for confounders prior to ligation, showed no association between ligation and adverse outcome (8, 9).

In the last decade, there has been a shift from aggressive pharmacological and/or surgical treatment toward a more conservative management (10). This change in policy can be justified by two arguments. First, since pharmacological treatment is not associated with an improvement in overall outcome (3, 4, 6, 11), fragile preterm infants are withheld from possible adverse effects of the pharmacological intervention. Second, there is a substantial rate of spontaneous closure of the DA (12, 13), even after failed pharmacological treatment (14). In summary, it is perceived that closure of the DA is delayed in preterm infants and treatment seems to only accelerate closure without improving outcome.

In this review and meta-analyses, we systematically reviewed the literature regarding mortality and morbidity associated with a conservative management for PDA in preterm and/or VLBW infants and compare most relevant outcome measures between RCTs and cohort studies.

## Methods

We performed a systematic literature review on 1 July 2020 in PubMed, EMBASE, and Cochrane library using the following search terms “preterm infant,” “very low birth weight infant,” “PDA,” “conservative treatment,” and “placebo” (Table 1). We excluded articles before 2000, as in this period antenatal corticosteroids and surfactant were not part of routine care. Reference lists of reviews and included articles were screened for additional studies. The Preferred Reporting Items for Systematic Reviews and Meta-Analyses (PRISMA) was followed (15). No review protocol was published.

**Table 1:** Search strategy.

**ID**	**Search**	**Hits**
**PubMed**		
#1	Ductus arteriosus, patent [MeSH Terms]	8,951
#2	Patent ductus arteriosus [Title/Abstract]	8,328
#3	Patency of the ductus arteriosus [Title/Abstract]	198
#4	Persistent ductus arteriosus [Title/Abstract]	490
#5	hsPDA [Title/Abstract]	190
#6	PDA [Title/Abstract]	12,050
#7	#1 OR #2 OR #3 OR #4 OR #5 OR #6	21,577
#8	Infant, extremely premature [MeSH Terms]	2,477
#9	Preterm [Title/Abstract]	75,235
#10	VLBW [Title/Abstract]	33,696
#11	Very low birth weight infant [Title/Abstract]	485
#12	Extremely premature infant [Title/Abstract]	145
#13	Premature birth [Title/Abstract]	3,607
#14	Prematurity [Title/Abstract]	21,967
#15	Infant, low birth weight [MeSH Terms]	34,180
#16	#8 OR #9 OR #10 OR #11 OR #12 OR #13 OR #14	114,537
#17	Conservative treatment [MeSH Terms]	2,955
#18	Conservative [Title/Abstract]	105,930
#19	Expectative [Title/Abstract]	141
#20	Expectant [Title/Abstract]	5,866
#21	Placebo [Title/Abstract]	214,408
#22	Placebos [MeSH Terms]	34,946
#23	No treatment [Title/Abstract]	30,200
#24	#17 OR #18 OR #19 OR #20 OR #21 OR #22 OR #23	365,730
#25	#7 AND #16 AND #24	214
#26	#25 with filters: Publication date from 01 Jan 2000	175
**EMBASE**		
#1	Patent ductus arteriosus.ti. or patent ductus arteriosus.ab.	9,985
#2	PDA.ti. or PDA.ab.	16,176
#3	Patency of the ductus arteriosus.ti. or patency of the ductus arteriosus.ab.	276
#4	Persistent ductus arteriosus.ti. or persistent ductus arteriosus.ab.	558
#5	hsPDA.ti. or hsPDA.ab.	162
#6	#1 OR #2 OR #3 OR #4 OR #5	22,852
#7	Extremely preterm infant.ti. or extremely preterm infant.ab.	161
#8	Extremely premature infant.ti. or extremely premature infant.ab.	156
#9	Extreme preterm infant.ti. or extreme preterm infant.ab.	15
#10	Extreme premature infant.ti. or extreme premature infant.ab.	17
#11	Very low birth weight.ti. or very low birth weight.ab.	9,275
#12	VLBW.ti. or VLBW.ab.	5,016
#13	Prematurity.ti. or prematurity.ab.	28,702
#14	Premature birth.ti. or premature birth.ab.	4,534
#15	#7 OR #8 OR #9 OR #10 OR #11 OR #12 OR #13 OR #14	42,047
#16	Conservative treatment.ti. or conservative treatment.ab.	39,060
#17	Conservative.ti. or conservative.ab.	137,261
#18	Expectative.ti. or expectative.ab.	214
#19	No treatment.ti. or no treatment.ab.	44,953
#20	Placebo.ti. or placebo.ab.	307,386
#21	Expectant.ti. or expectant.ab.	7,805
#22	#16 OR #17 OR #18 OR #19 OR #20 OR #21	489,991
#23	#6 AND #15 AND #22	117
#24	Limit #23 to year = “2000–current”	93
**Cochrane library**		
#1	MeSH descriptor: [Ductus Arteriosus, Patent] explode all trees	285
#2	(Patent ductus arteriosus):ti,ab	750
#3	(PDA):ti,ab	959
#4	(Persistent ductus arteriosus):ti,ab	92
#5	(hsPDA):ti,ab	33
#6	(Patency of the ductus arteriosus):ti,ab	34
#7	(#6 OR #5 OR #4 OR #3 OR #2 OR #1)	1,403
#8	(Extremely preterm):ti,ab	564
#9	MeSH descriptor: [Infant, Extremely Premature] explode all trees	177
#10	MeSH descriptor: [Infant, Low Birth Weight] explode all trees	2,157
#11	(Preterm):ti,ab	13,188
#12	(VLBW):ti,ab	896
#13	(Very low birth weight infants):ti,ab	1,680
#14	(Infant, Extremely Premature):ti,ab	103
#15	(Premature birth):ti,ab	2,672
#16	(Prematurity):ti,ab	2,349
#17	#9 OR #10 OR #11 OR #12 OR #13 OR #14 OR #15 OR #16	17,085
#18	MeSH descriptor: [Conservative Treatment] explode all trees	119
#19	(Conservative):ti,ab	8,311
#20	(Expectative):ti,ab	90
#21	(Expectant):ti,ab	1,097
#22	(Placebo):ti,ab	293,798
#23	(No treatment):ti,ab	262,232
#24	MeSH descriptor: [Placebos] explode all trees	23,914
#25	#18 OR #19 OR #20 OR #21 OR #22 OR #23 OR #24	489,460
#26	#7 AND #17 AND #25	339
#27	#26 with Cochrane Library publication date between Jan 2000 and 1 Jul 2020	282

Articles were included when it concerned (a) preterm infants <32 weeks' gestation or VLBW infants <1,500 g, with a PDA (irrespective of diagnostic criteria) in (b) a RCT or cohort study with (c) at least one study group managed conservatively (defined as <25% open-label pharmacological treatment with ibuprofen, indomethacin, or paracetamol and/or ligation/endovascular closure for RCTs and <25% active treatment during follow-up for cohort studies) as our aim is to compare active treatment with conservative management instead of “delayed” treatment and when (d) data about the primary outcome (mortality) or secondary outcome measures (BPD, NEC, IVH, and ROP) were available. Conservative management was defined as the absence of any pharmacological or surgical/endovascular intervention with the intention to actively close the DA other than fluid restriction, diuretics, and/or ventilator adjustments.

Articles were excluded if: (a) data about outcome measurements were not available per treatment regimen; (b) language was not English, German or Dutch; and (c) the paper was a conference abstract.

Two reviewers (TH and EJ) screened the title and abstract of the retrieved papers. Disagreements were resolved by a third reviewer (WdB). For eligible studies, corresponding authors were contacted for missing data from subgroups. Two reviewers (TH and EJ) assessed the risk of bias with the Cochrane Risk of Bias Tool for included RCTs (16) and the Newcastle-Ottawa Scale (NOS) for cohort studies (17). Disagreements were resolved by a third reviewer (WdB).

Data extraction regarding study design, study population, definition of (hs)PDA (not specified, clinical parameters only, echocardiographic parameters only, or both clinical and echocardiographic parameters), definition of conservative management (respiratory adjustments, fluid restriction and/or diuretics, or no pharmacological/surgical PDA treatment), percentage open-label active treatment (pharmacological and/or surgical) in the conservative management group, and outcome parameters (mortality, BPD, NEC, IVH, and ROP) from included studies were done by two reviewers (TH and EJ).

If available from the cohort studies, adjusted odds ratio (aOR) was also extracted and expressed as conservative management group compared with the active treatment regimen.

Conservative management was compared with five active treatment regimens, namely (1) any active treatment, defined as treatment with either ibuprofen, indomethacin, or paracetamol and/or ligation/endovascular closure; (2) any pharmacological treatment, defined as treatment with ibuprofen, indomethacin, and/or paracetamol, both prophylactic and non-prophylactic; (3) non-prophylactic pharmacological treatment, defined as treatment with ibuprofen, indomethacin, and/or paracetamol beyond a postnatal age of 24 h; (4) prophylactic pharmacological treatment, defined as treatment with ibuprofen, indomethacin, or paracetamol within a postnatal age of 24 h irrespective of PDA status; and (5) ligation/endovascular closure, defined as ligation/endovascular closure without preceding pharmacological treatment.

Some studies included a subgroup without a PDA. Those subgroups were excluded from the initial analysis, but in a subgroup analysis, we included those low-risk patients in the conservative treatment group to investigate their modulating effect on outcome measures. Furthermore, our main inclusion criterium for PDA was irrespective of diagnostic criteria used. In a subgroup analysis, we will only include studies with an echocardiographically confirmed PDA >1.5 mm in both subgroups.

Outcome measures were entered in Review Manager Software for meta-analysis (Revman version 5.3 Copenhagen: The Nordic Cochrane Center, The Cochrane Collaboration, 2014). Meta-analysis was performed for RCTs and cohort studies separately per defined treatment regimen. We used random effect if the heterogeneity (*I*^2^) was >50% (18) and fixed effect in case of a lower heterogeneity. Effects are presented as risk ratio (RR) and risk difference (RD) with 95% confidence interval (95% CI).

The methodological quality of the studies' outcome parameters was examined with the GRADE method (19). We assessed imprecision as serious if the total number of events was <300 or if the width of the CI of the RR was >0.25. We used the GRADE-pro GDT 2016 software (GRADEpro Guideline Development Tool [Software], McMaster University, 2015) to create a “summary of findings” table to report the quality of evidence. The GRADE approach results in an assessment of the evidence in one of four grades of evidence: high, moderate, low, or very low certainty.

## Results

**Figure 1:**
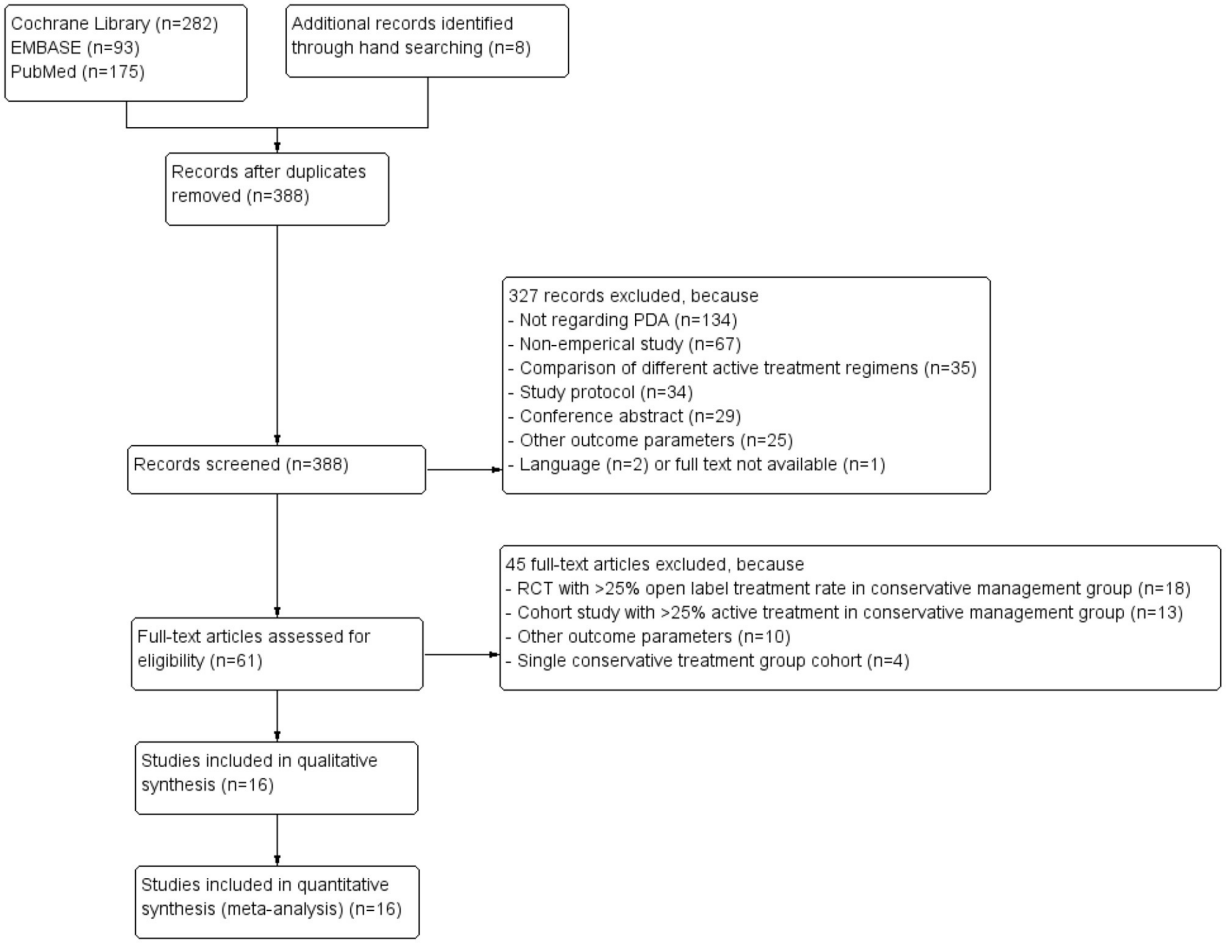
Preferred Reporting Items for Systematic Reviews and Meta-Analysis (PRISMA) flow diagram of systematic literature review (15).

Our search revealed 388 unique articles, of which four RCTs (20–23) and 12 cohort studies (7, 24–34) could be included in the meta-analyses. Figure 1 depicts the PRISMA flow diagram showing the retrieval process of the included articles (15). Due to our inclusion criterium of strict conservative management, we had to exclude many RCTs (*n* = 18) because of >25% open-label active treatment and cohort studies (*n* = 13) because of >25% active treatment during follow-up in the conservative management arm.

### Study Characteristics

A total of 63,254 patients were analyzed, of which 720 patients from RCTs and 41,804 from cohort studies were included in the initial meta-analyses. The remaining (*n* = 20,730) were classified as a subgroup without a PDA in four cohort studies and did not receive any (prophylactic) treatment (26, 29) and were therefore excluded from the initial analyses.

In Table 2, the characteristics of the included RCTs and cohort studies are shown. The used definitions for a (hs)PDA varied extensively between studies. Patient characteristics are presented in Table 3.

**Table 2:** Study characteristics of included studies.

	**References**	**Period**	**Design**	**Treatment strategies**	**Main inclusion criteria**	**Main exclusion criteria**	**Infants (*n*)**	**Conservative management**	**Used drug(s) (dosage if available)**	**(hs)PDA diagnosis**	**hsPDA definition**
RCTs	Härkin et al. (20)	2013 2015	Double-blind Monocenter	CONS (placebo) vs. PPT	GA <32 weeks	CM, lethal disease, PPHN	48	Placebo (0.45% saline)	Paracetamol (once 20 mg/kg; 24 × 7.5 mg/kg every 6 h)	CLIN + ECHO	CLIN: increased respiratory support, decreased blood pressure, increased pulse pressure, pulmonary congestion, cardiomegaly, hepatomegaly, murmur, hyperdynamic precordium, bounding pulses ECHO: LA/Ao >1.4, PDA diameter/LPA >1.5, large LtR shunt
	Kumar Nair et al. (21)	1998 2001	Non-blinded Monocentre	CONS vs. PPT	BW 750–1,250 g, absence of IVH prior to inclusion	GA <26 weeks, AS^5^ <5, CM, sepsis, contraindication for PPT	115	Not defined	INDO (3 × 0.1 mg/kg/day) start 6–12 h PNA	CLIN + ECHO	CLIN: hemorrhagic pulmonary edema, cardiomegaly on chest X-ray or failure of weaning from ventilatory support ECHO: not defined
	Sung et al. (22)	2014 2019	Double-blinded Monocenter	CONS (placebo) vs. PT	GA 23–30-weeks with respiratory support and PDA at PNA 6–14 days	CHD, life threatening CM, predominant RtL shunt, IVH ≥3, contraindication for PT	142	Placebo (0.9% saline)	IBU p.o. (10–5–5 mg/kg)	ECHO	ECHO: diameter >1.5 mm with predominant LtR shunt
	Van Overmeire et al. (23)	1999 2001	Double-blind Multicenter	CONS (placebo) vs. PPT	GA 24–30 weeks within 6 h PNA	Major CM, IVH > grade I, AS^5^ <5, sepsis, hypotension, contraindication for PPT	415	Placebo	IBU (10–5–5 mg/kg)	Not defined	Not defined
Cohort studies	Alexander et al. (24)	1996 2005	Retrospective (chart review) Monocentre	CONS vs. PT or LIG or LIG after PT	BW <1,000 g with ECHO of PDA documented	Not defined	298	No PT and/or LIG	INDO (3 × 0.2 mg/kg every 12 h)	ECHO	Not defined
	Bourgoin et al. (25)	2003 2011	Prospective Multicenter	CONS for (non-hs)PDA vs. PT or LIG for hsPDA	Discharged alive	Dead <2 years, CM, lost to follow-up	857	No PT and/or LIG	IBU (10–5–5 mg/kg)	ECHO	PDA diameter >1.5 mm; LA/Ao >1.5; pulsatile flow in the DA; retrograde/absent diastolic flow in the cerebral anterior artery or in the descending thoracic aorta.
	Härkin et al. (26)	2005 2013	Retrospective (FMBR database) Multicentre	CONS vs. PT and/or LIG or no PDA	GA <32 weeks	Mortality <7 days PNA, severe CM	3,668	No PT and/or LIG	INDO or IBU	CLIN + ECHO	CLIN: murmur, hyperdynamic precordium, bounding pulses, increased need for respiratory support and increased pulse pressure ECHO: not defined
	Heuchan et al. (27)	2005 2009	Retrospective Monocentre	CONS vs. LIG or no PDA	Echocardiogram performed at PNA 6–48 h	CHD	25	No PT and/or LIG	None (primary LIG)	CLIN + ECHO	CLIN: murmur, hypotension, pulmonary hemorrhage, renal impairment ECHO: color Doppler diameter
	Laughon et al. (28)	1997 2004	Retrospective Multicenter	CONS vs. PPT or STG or LIG or no PDA	GA 23–30-weeks	Not defined	34,602	No PT and/or LIG	INDO	Not defined	Not defined
	Letshwiti et al. (29)	2004 2011	Retrospective Monocenter	CONS vs. PT (subdivided in ETG and STG) or no PDA	BW <1,500 g	Not defined	371	PEEP ≥5 cmH_2_O, FR (130–150 ml/kg/day)	IBU (10–5–5 mg/kg)	ECHO	PDA diameter >2 mm; LA/Ao >1.5; evidence of reduced splanchnic Doppler flow
	Lokku et al. (30)	2006 2012	Retrospective (CNN database) Multicentre	CONS vs. PT and/or LIG	GA 23–32-weeks with CLIN and/or ECHO of PDA	Dead <72 h PNA, severe CM, ≥triplet, missing data regarding date of birth or sex	5,824	No PT and/or LIG	INDO	CLIN ± ECHO	CLIN: systolic murmur, bounding pulses, wide pulse pressure ECHO: not defined
	Mirea et al. (7)	2004 2008	Retrospective (CNN database) Multicenter	CONS vs. PT and/or LIG	GA ≤ 32 weeks with CLIN and/or ECHO of PDA	Dead <72 h PNA, CHD	3,556	FR and/or diuretics	INDO	CLIN ±- ECHO	Not defined
	Mohamed et al. (31)	2001 2014	Retrospective (database) Monocentre	CONS vs. PT	BW <1,500 g	Not defined	643	Standard respiratory setting, no FR	INDO [2001–2006] IBU [2006–2014]	CLIN + ECHO	CLIN: cardiac murmur, pulsating pericardium, wide peripheral pulses,
		2014									increasing metabolic acidosis (base excess < -8 mEq/L) ECHO: moderate to severe PDA
	Sadeck et al. (32)	2010 2011	Retrospective (BNRN database) Multicentre	CONS vs. PT and/or LIG	BW <1,000 g, GA <33 weeks with ECHO of PDA	Died or transferred <3 days PNA, congenital infections, CM	494	No PT and/or LIG	INDO/IBU	Not defined	Not defined
	Slaughter et al. (33)	2006 2013	Retrospective (PHIS database) Multicentre	CONS vs. PT	GA <28 weeks	Hospitalized <3 days, admitted >24 h PNA, no recorded discharge status	12,018	No PT and/or LIG	INDO/IBU	Not defined	Not defined
	Sung et al. (34)	2009 2014	Retrospective (database) Monocentre	CONS vs. PT and/or LIG	GA 23–26- weeks	Died <48 h, CHD, PDA <2 mm or off ventilator	178	FR and diuretics if indicated	INDO (3 × 0.2 mg/kg every 12 h)	CLIN + ECHO	CLIN: deterioration in respiratory condition, cardiac murmur, hyperactive precordium,
											hypotension and widened pulse pressure ECHO: PDA diameter ≥2 mm with predominant LtR shunt

*AS^5^, Apgar score at 5 min postpartum; BNRN, Brazilian Neonatal Research Network; BW, birth weight; CHD, congenital heart disease; CM, congenital malformation and/or chromosomal abnormality and/or genetic syndrome; CNN, Canadian Neonatal Network; CLIN, clinical diagnosis; CONS, conservative management; d, days; DA, ductus arteriosus; ECHO, echocardiographic parameters; ETG, early treatment group; FMBR, Finnish Medical Birth Register; FR, fluid restriction; GA, gestational age; IBU, ibuprofen; INDO, indomethacin; IVH, intraventricular hemorrhage; LA/Ao, left atrium to aortic root ratio; LIG, ligation; LPA, left pulmonary artery; LtR, left to right; (hs)PDA, (hemodynamically significant) patent ductus arteriosus; PEEP, positive end expiratory pressure; PHIS, Pediatric Health Information System; PNA, postnatal age; PPHN, persistent pulmonary hypertension of a newborn; PT, pharmacological treatment; PPT, prophylactic pharmacological treatment; RCTs, randomized controlled trials; RtL, right to left; STG, standard treatment group*.

**Table 3:** Patient characteristics of included studies.

		**Patient characteristics**															
		**Conservative management**				**Prophylactic pharmacological treatment**			**Pharmacological treatment**			**Ligation closure**			**Pharmacotherapy and/or ligation closure**		
	**References**	**Infants (male/*N*)**	**GA (weeks)**	**BW (grams)**	**Open-label treatment**	**Infants (male/N)**	**GA (weeks)**	**BW (g)**	**Infants (male/N)**	**GA (weeks)**	**BW (g)**	**Infants (male/N)**	**GA (weeks)**	**BW (g)**	**Infants (male/N)**	**GA (weeks)**	**BW (g)**
RCTs	Härkin et al. (20)	14/25	28.3 ± 2.06	1,120 ± 340	12.0%	13/23	28.4 ± 2.36	1,220 ± 430	–	–	–	–	–	–	–	–	–
	Kumar Nair et al. (21)	NA/59	27.9 ± 1.4	995 ± 83.6	NA	NA/56	27.8 ± 1.2	989.5 ± 93.5	–	–	–	–	–	–	–	–	–
	Sung et al. (22)	41/72	26.7 ± 2.0	915 ± 243	0%	–	–	–	28/70	26.8 ± 2.1	893 ± 256	–	–	–	–	–	–
	Van Overmeire et al. (23)	NA/210	28.1 ± 1.6	1,065 ± 324	24.8%	NA/205	28.1 ± 1.7	1,048 ± 315	–	–	–	–	–	–	–	–	–
Cohort studies	Alexander et al. (24)	NA/54	25.7 ± 1.9	729.6 ± 169.6	0%	–	–	–	?/198	**26.1 ± 1.9**	**739 ± 140.5**	?/46	**24.8 ± 1.5**	**678.7** ± **153.5**	–	–	–
	Bourgoin et al. (25)	254/505	NA	977 ± 212	0%	–	–	–	134/248	NA	**911 ± 191**	40/104	NA	**833 ± 225**	–	–	–
	Härkin et al. (26)	98/182	28.82 ± 2.41	1,225 ± 402	0%	–	–	–	395/770	**26.3** ± **1.2**	**1,115** ± **336**	66/112	**25.6** ± **1.4**	**834** ± **297**	134/250	**25.5** ± **1.3**	**846** ± **278**
	Heuchan et al. (27)	4/7	27 [25–28]	1,046 [680–1,440]	0%	–	–	–	–	–	–	8/11	26 [24–27]	780 [613–1,240]	–	–	–
	Laughon et al. (28)	2,201/ 3,886	27 [26–29]	970 [750–1,220]	0%	3,293/ 6,189	**26 [25–28]**	873 [703–1075]	3,021/ 5,690	27 [25–29]	**960 [760–1,205]**	388/701	**25 [24–27]**	**730 [624–895]**	–	–	–
	Letshwiti et al. (29)^§^	16/34	27.4 ± 2.7	1,010 ± 250	14.7%	–	–	–	**15/52**	27.9 ± 2.0	1,040 ± 270	–	–	–	–	–	–
									26/52	27.5 ± 1.9	1,010 ± 280						
	Lokku et al. (30)^†^	811/ 1,486	28.2 ± 2.3	NA	0%	–	–	–	1,754/ 3,226	**27.1** ± **2.1**	NA	165/280	**26.0** ± **2.2**	NA	423/832	**25.6** ± **1.7**	NA
			28.2 ± 2.4	NA						**26.6** ± **2.0**	NA		**26.0** ± **1.8**	NA		**25.3** ± **1.6**	NA
	Mirea et al. (7)	321/577	28.3 ± 2.3	NA	0%	–	–	–	1,062/ 2,026	**27.0** ± **2.1**	NA	185/327	**26.0** ± **2.3**	NA	325/626	**25.5** ± **1.7**	NA
	Mohamed et al. (31)	122/228	28.0 ± 3.4	1,016 ± 340	0%	–	–	–	216/415	27.7 ± 2.9	988 ± 311	–	–	–	–	–	–
	Sadeck et al. (32)	91/187	27.6 ± 2.2	772 ± 142.3	0%	–	–	–	90/205	27.4 ± 1.9	**804 ± 121.6**	48/102	**26.6** ± **1.8**	781 ± 118.5	–	–	–
	Slaughter et al. (33)	4,302/ 8,130	NA	NA	0%	–	–	–	2,068/ 3,888	NA	NA	–	–	–	–	–	–
	Sung et al. (34)	54/97	24.5 ± 1.0	718 ± 137	0%	–	–	–	–	–	–	35/81	24.6 ± 1.1	728 ± 134	–	–	–

*Data presented as number, percentage, mean ± standard deviation or median [interquartile range]. Statistical difference between other groups in same study are bold*.

*ETG; early treatment group, GA; gestational age, N; total infants, NA; not available, PDA; patent ductus arteriosus, RCTs; randomized controlled trials, STG; symptomatic treatment group*.

§ *Pharmacological treatment in study subdivided between early treatment and symptomatic treatment*.

† *Cohort subdivided in cohort 2006–2008 and 2009–2012. BW; birth weight*.

Three RCTs were placebo controlled (20, 22, 23), while for one RCT, the control arm was not specified (21). In the cohort studies, the definition of conservative management ranged from no treatment at all to a regimen with fluid restriction, diuretics, and/or adaptation in ventilator settings or the absence of any pharmacological/surgical treatment.

Mortality was heterogeneously defined, since four studies excluded early neonatal deaths within 24–72 postnatal hours (7, 30, 32, 34). The outcome parameter BPD was defined according to the international criteria at 36 weeks postmenstrual age in 12 papers (7, 20, 22, 26–34). NEC was defined according to the Bell stadium in nine studies (7, 20, 22, 23, 26, 27, 30, 31, 34). Thirteen studies defined IVH as grade III or higher (7, 20–23, 25–32, 34), ROP was defined as stage 3 or higher in five studies (7, 22, 26, 28, 34). No study described endovascular closure.

### Risk of Bias

The quality of the included RCTs was high, given the low risk of bias (Table 4). The quality of the cohort studies was classified as moderate (Table 5).

**Table 4:** Risk of bias assessment of included randomized controlled trials according to the cochrane risk of bias tool (16).

	**Härkin et al. (20)**	**Kumar Nair et al. (21)**	**Sung et al. (22)**	**Van Overmeire et al. (23)**
Adequate sequence generation	+	+	+	+
Allocation concealment	+	+	+	+
Performance bias	+	–	+	+
Detection bias	+	?	+	+
Attrition bias	+	+	+	+
Reporting bias	+	+	+	+
Other bias	?	?	?	?

**Table 5:** Risk of bias assessment of included cohort studies according to Newcastle–Ottawa scale (NOS) (17).

**References**	**Selection**				**Comparability**	**Outcome**		
	**A**	**B**	**C**	**D**	**E**	**F**	**G**	**H**
Alexander et al. (24)	*	*	*	*	**	*	–	*
Bourgoin et al. (25)	*	*	*	*	**	*	*	*
Härkin et al. (26)	*	*	*	*	**	*	*	*
Heuchan et al. (27)	*	*	*	*	–	*	–	*
Laughon et al. (28)	–	*	*	*	**	*	–	*
Letshwiti et al. (29)	*	*	*	*	–	*	–	*
Lokku et al. (30)	*	*	*	*	*	*	–	*
Mirea et al. (7)	*	*	*	*	**	*	*	*
Mohamed et al. (31)	*	*	*	*	**	*	*	*
Sadeck et al. (32)	*	*	*	*	–	*	–	*
Slaughter et al. (33)	*	*	*	*	*	*	*	*
Sung et al. (34)	*	*	*	*	**	*	–	*

*A; representatives of the exposed cohort, B; selection of non-exposed cohort, C; ascertainment of exposure, D; demonstration that outcome of interest was not present at start of study, E; comparability of cohorts on the basis of the design or analysis, F; assessment of outcome, G; was follow-up long enough for outcomes to occur, H; adequacy of follow-up of cohorts*.

Maximum score for each item is one star (*), except for comparability for which two stars (**) can be scored, and if not scored positive presented as “–”.

### Meta-Analysis

#### Meta-Analysis Outcome Measures Randomized Controlled Trials

Meta-analysis of the four included RCTs did not show significant differences for mortality or morbidity in any of the predefined groups, as is shown in Table 6 (Supplementary Material 1). The quality of the evidence was graded as moderate to low (Supplementary Material 2).

**Table 6:** Outcome measurements after meta-analysis.

**Comparison**	**(Sub)group**	**Mortality**	**BPD (any definition)**	**NEC (any stage)**	**IVH (any grade)**	**ROP (any stage)**
Conservative management vs. any active treatment	RCTs	1.09^§^ (0.73–1.61)	0.80 (0.55–1.17)	1.17^§^ (0.65–2.13)	1.00^§^ (0.71–1.40)	0.85^§^ (0.45–1.60)
		*0.01 (−0.04 to 0.06)*	*−0.06 (−0.16 to 0.04)*	*0.01 (−0.02 to 0.04)*	*0.00 (−0.04 to 0.05)*	*−0.03 (−0.13 to 0.08)*
		[4; 720]	[4; 709]	[4; 720]	[4; 720]	[2; 190]
	Cohort	**1.34 (1.12–1.62)**	**0.55 (0.46–0.65)**	**0.85**^**§**^ **(0.77–0.93)**	**0.88**^§^ **(0.83–0.95)**	**0.47 (0.28–0.79)**
	studies	***0.03 (0.01–0.06)***	***−0.18 (−0.24 to −0.12)***	***−0.01 (−0.02 to −0.01)***	***−0.02 (−0.03 to −0.01)***	***−0.06 (0.10 to −0.02)***
		**[11; 40,916]**	**[11; 39,993]**	**[9; 28,004]**	**[10; 28,504]**	**[8; 26,608]**
Conservative management vs. any pharmacological treatment	RCTs	1.09^§^ (0.73–1.61)	0.80 (0.55–1.17)	1.17^§^ (0.65–2.13)	1.00^§^ (0.71–1.40)	0.85^§^ (0.45–1.60)
		*0.01 (−0.04 to 0.06)*	*−0.06 (−0.16 to 0.04)*	*0.01 (−0.02 to 0.04)*	*0.00 (−0.04 to 0.05)*	*−0.03 (−0.13 to 0.08)*
		[4; 720]	[4; 709]	[4; 720]	[4; 720]	[2; 190]
	Cohort	**1.46 (1.14–1.85)**	**0.63 (0.51–0.78)**	1.06 (0.78–1.46)	0.95^§^ (0.89–1.02)	**0.57 (0.40–0.82)**
	studies	***0.05 (0.01–0.08)***	***−0.12 (−0.17 to −0.06)***	*0.01 (−0.02 to 0.03)*	*−0.01 (−0.02 to 0.00)*	***−0.04 (−0.06 to −0.02)***
		**[7; 24,729]**	**[7; 24,104]**	[6; 23,965]	[7; 24,446]	**[5; 22,892]**
Conservative management vs. nonprophylactic pharmacological treatment	RCTs	0.97^§^ (0.33–2.87)	0.89^§^ (0.60–1.32)	0.42^§^ (0.11–1.55)	1.94^§^ (0.37–10.28)	0.91^§^ (0.47–1.74)
		*0.00 (−0.09 to 0.09)*	*−0.05 (−0.22 to 0.12)*	*−0.06 (−0.14 to 0.03)*	*0.03 (−0.04 to 0.09)*	*−0.02 (−0.15 to 0.11)*
		[1; 142]	[1; 131]	[1; 142]	[1; 142]	[1; 142]
	Cohort	**1.54 (1.13–2.09)**	**0.60 (0.48–0.76)**	1.01 (0.81–1.26)	0.97^§^ (0.90–1.04)	**0.57 (0.37–0.87)**
	studies	***0.04 (0.00–0.07)***	***−0.13 (−0.18 to −0.07)***	*0.00 (−0.02 to 0.01)*	*0.00 (−0.02 to 0.01)*	***−0.04 (−0.06 to −0.01)***
		**[6; 18,148]**	**[6; 17,779]**	[5; 17,640]	[6; 18,141]	**[4; 16,567]**
Conservative management vs. prophylactic pharmacological treatment	RCTs	1.11^§^ (0.72–1.69)	0.66 (0.25–1.76)	1.63^§^ (0.81–3.31)	0.96^§^ (0.68–1.35)	0.31^§^ (0.01–7.20)
		*0.01 (−0.04 to 0.07)*	*−0.06 (−0.19 to 0.07)*	*0.03 (−0.01 to 0.07)*	*−0.01 (−0.06 to 0.05)*	*−0.04 (−0.15 to 0.07)*
		[3; 578]	[3; 578]	[3; 578]	[3; 578]	[1; 48]
	Cohort	0.92^§^ (0.83–1.01)	**0.82**^§^ **(0.78–0.87)**	**0.77**^§^ **(0.67–0.88**)	0.91^§^ (0.81–1.01)	**0.66**^§^ **(0.59–0.75)**
	studies	*−0.01 (−0.03 to 0.00)*	***−0.07 (−0.09 to −0.05)***	***−0.02 (−0.03 to −0.01)***	*−0.01 (−0.03 to 0.00)*	***−0.05 (−0.06 to −0.03)***
		[1; 10,075]	**[1; 9,580]**	**[1; 9,580]**	[1; 9,580]	**[1; 9,580]**
Conservative management vs. ligation	RCTs	NA	NA	NA	NA	NA
	Cohort	1.25 (0.76–2.05)	**0.36 (0.27–0.47)**	**0.49 (0.35–0.68)**	**0.65 (0.48–0.88)**	**0.23 (0.18–0.31)**
	studies	*0.07 (−0.02 to 0.16)*	***−0.36 (−0.47 to −0.26)***	***−0.07 (−0.12 to −0.02)***	***−0.09 (−0.15 to −0.03)***	***−0.17 (−0.28 to −0.07)***
		[7; 7,867]	**[7; 8,021]**	**[6; 7,535]**	**[7; 8,020]**	**[6; 7,146]**

*Data presented as risk ratio (95% confidence interval) and risk difference (95% confidence interval) after random effect unless otherwise specified, [number of studies; number of patients]*.

*BPD; bronchopulmonary dysplasia, IVH; intraventricular hemorrhage, NA; not available, NEC; necrotizing enterocolitis, RCTs; randomized controlled trials, ROP; retinopathy of prematurity*.

§ *Fixed effect. Significant differences are depicted in bold fonts. To show difference between risk ratio with 95% confidence interval in non-italic font, risk difference with 95% confidence interval in italic and number of studies/patients within brackets*.

#### Meta-Analysis Outcome Measures Cohort Studies

Meta-analysis of the cohort studies revealed that conservative management was associated with a higher risk for mortality compared with any active treatment (RR, 1.34 [1.12–1.62]; RD, 0.03 [0.01–0.06]), any pharmacological treatment (RR, 1.46 [1.14–1.85]; RD, 0.05 [0.01–0.08]), and non-prophylactic pharmacological treatment (RR, 1.54 [1.13–2.09]; RD, 0.04 [0.00–0.07]) (Table 6; Supplementary Material 3). The quality of the evidence was graded as very low (Supplementary Material 4).

Conservative management was associated with a lower risk for BPD and ROP compared with both each separate active treatment regimen and any active treatment. The risk for NEC was significantly lower for conservative management in comparison with any active treatment (RR, 0.85 [0.77–0.93]; RD, −0.01 [−0.02 to −0.01]), prophylactic pharmacological treatment (RR, 0.77 [0.67–0.88]; RD, −0.02 [−0.03 to −0.01]), and ligation (RR, 0.49 [0.35–0.68]; RD, −0.07 [−0.12 to −0.02]). Conservative management was associated with a lower risk for IVH compared with any active treatment (RR, 0.88 [0.83–0.95]; RD, −0.02 [−0.03 to 0.01]) and ligation (RR, 0.65 [0.48–0.88]; RD, −0.09 [−0.15 to −0.03]) (Table 6; Supplementary Material 3). The quality of the evidence was graded as very low (Supplementary Material 4).

#### Meta-Analysis Outcome Measures Cohort Studies Including Patients Without a PDA

Subgroup baseline characteristics and outcome measures were available for patients without a PDA in three studies (*n* = 20,497) (26–28). In this subgroup, analysis outcome of those patients was added to the conservative management group (Table 7). The higher risk for mortality lost significance in almost all subgroups, while the lower risk for morbidity was even more pronounced (Table 8, Supplementary Material 5).

**Table 7:** Patient characteristics of cohort studies with subgroup without patent ductus arteriosus.

	**Patient characteristics**					
	**Conservative management**			**No or asymptomatic PDA**		
**References**	**Infants (male/*N*)**	**GA (weeks)**	**BW (grams)**	**Infants (male/N)**	**GA (weeks)**	**BW (g)**
Härkin et al. (26)	98/182	28.82 ± 2.41	1,225 ± 402	1,398/2,536^†^	**29.7 ± 1.9**	**1,340 ± 371**
Heuchan et al. (27)	4/7	27 [25–28]	1,046 [680–1,440]	3/7	26 [24–28]	912 [500–1,440]
Laughon et al. (28)	2,201/3,886	27 [26–29]	970 [750–1,220]	9,796/18,136	**29 [27–30]**	**1,170 [895–1,400]**

*Data presented as number, mean ± standard deviation or median [interquartile range]. Statistical difference between other treatment groups in same study are in bold*.

*BW; birth weight, GA; gestational age, N; total infants, PDA; patent ductus arteriosus*.

*Including 182 patients with PDA that were conservatively managed and analyzed separately*.

**Table 8:** Outcome measurements after meta-analysis of cohort studies including patient without patent ductus arteriosus.

**Comparison**	**Mortality**	**BPD (any definition)**	**NEC (any stage)**	**IVH (any grade)**	**ROP (any stage)**
Conservative management vs. any active treatment	1.14 (0.91–1.43) *0.02 (−0.01 to 0.04)* [11; 61,372]	**0.47 (0.38–0.57)** ***−0.20 (−0.26 to −0.14)***** [11; 57,400]**	**0.78 (0.61–0.99)** ***−0.02 (−0.04 to 0.00)***** [9; 45,831]**	**0.71 (0.50–0.99)** ***−0.04 (−0.07 to −0.01)***** [10; 46,266]**	**0.34 (0.27–0.43)** ***−0.07 (−0.10 to −0.03)***** [8; 44,371]**
Conservative management vs. any pharmacological treatment	1.20 (0.94–1.54) *0.02 (−0.01 to 0.04)* [7; 45,178]	**0.51 (0.42–0.61)** ***−0.15 (−0.20 to −0.10)***** [7; 41,504]**	0.92 (0.63–1.35) *0.00 (−0.03 to 0.02)* [6; 41,785]	0.77 (0.53–1.12) *−0.02 (−0.05 to 0.01)* [7; 42,221]	**0.39 (0.33–0.46)** ***−0.05 (−0.09 to −0.01)***** [5; 40,648]**
Conservative management vs. non-prophylactic pharmacological treatment	1.20 (0.97–1.49) *0.01 (−0.01 to 0.04)* [6; 38,597]	**0.47 (0.39–0.57)** ***−0.16 (−0.22 to −0.10)***** [6; 35,179]**	0.83 (0.60–1.15) *−0.01 (−0.04 to 0.01)* [5; 35.460]	0.72 (0.50–1.05) *−0.03 (−0.06 to 0.00)* [6; 35,896]	**0.42 (0.33–0.53)** ***−0.05 (−0.09 to −0.02)***** [4; 32,138]**
Conservative management vs. prophylactic pharmacological treatment	**0.74 (0.69–0.79)** ***−0.04 (−0.05 to −0.03)***** [1; 28,211]**	**0.54 (0.51–0.56)** ***−0.19 (−0.20 to −0.17)***** [1; 25,151]**	**0.59 (0.53–0.65**) ***−0.04 (−0.05 to −0.03)***** [1; 25,151]**	**0.44 (0.40–0.48)** ***−0.07 (−0.08 to −0.07)***** [1; 25,151]**	**0.35 (0.32–0.39)** ***−0.09 (−0.10 to −0.08)***** [1; 25,151]**
Conservative management vs. ligation	0.96 (0.49–1.87) *0.05 (−0.05 to 0.14)* [7; 28,323]	**0.30 (0.20–0.43)** ***−0.39 (−0.49 to −0.28)***** [7; 25,428]**	**0.40 (0.28–0.57)** ***−0.09 (−0.14 to −0.03)***** [6; 25,410]**	**0.49 (0.25–0.94)** ***−0.11 (−0.20 to −0.03)***** [7; 25,782]**	**0.16 (0.14–0.18)** ***−0.18 (−0.30 to −0.06)***** [6; 24,909]**

*Data presented as risk ratio (95% confidence interval) and risk difference (95% confidence interval) after random effect, [number of studies; number of patients]. Significant differences are depicted in bold fonts*.

*BPD; bronchopulmonary dysplasia, IVH; intraventricular hemorrhage, NEC; necrotizing enterocolitis, RCTs; randomized controlled trials, ROP; retinopathy of prematurity. To show difference between risk ratio with 95% confidence interval in non-italic font, risk difference with 95% confidence interval in italic and number of studies/patients within brackets*.

#### Meta-Analysis Outcome Measures Cohort Studies With Echocardiographic Defined PDA

We performed a subgroup meta-analysis on the two cohort studies that used echocardiographic definitions (*n* = 316) (29, 34). Outcome measurements, as presented in Table 9, showed a significant lower risk for BPD in the conservative treated group compared with the available subgroups any treatment and any/non-prophylactic pharmacological treatment. Mortality and other morbidity outcomes showed no difference.

**Table 9:** Outcome measurements after meta-analysis of cohort studies including echocardiographic defined patent ductus arteriosus.

**Comparison**	**Mortality**	**BPD(any definition)**	**NEC (any stage)**	**IVH(any grade)**	**ROP (any stage)**
Conservative management versus any active treatment	0.79 (0.35–1.78)	**0.43 (0.32 to 0.58)**	0.94 (0.47 to 1.88)	0.66 (0.36 to 1.22)	1.17 (0.39 to 3.54)
	*−0.02 (−0.08 to 0.04)*	–***0.38 (**–**0.49 to** –**0.27)***	*−0.01 (−0.08 to 0.06)*	*−0.06 (−0.14 to 0.02)*	*0.01 (−0.06 to 0.08)*
	[2; 316]	**[2; 287]**	[2; 316]	[2; 316]	[1; 178]
Conservative management versus any pharmacological treatment/non -prophylactic pharmacological treatment	0.61 (0.07 to 5.06)	**0.38 (0.18 to 0.80)**	0.76 (0.17 to 3.43)	0.66 (0.20 to 2.14)	NA
	*−0.02 (−0.09 to 0.05)*	–***0.30 (**–**0.47 to** –**0.14)***	*−0.02 (−0.11 to 0.08)*	*−0.05 (−0.16 to 0.07)*	
	[1; 138]	**[1; 132]**	[1; 138]	[1; 138]	

*Data presented as risk ratio (95% confidence interval) and risk difference (95% confidence interval) after fixed effect, [number of studies; number of patients]. Significant differences are depicted in bold fonts. BPD, bronchopulmonary dysplasia; IVH, intraventricular haemorrhage; NA, not available; NEC, necrotizing enterocolitis; RCTs, randomised controlled trials; ROP, retinopathy of prematurity*.

*To show difference between risk ratio with 95% confidence interval in non-italic font, risk difference with 95% confidence interval in italic and number of studies/patients within brackets*.

### Adjusted Outcome

#### Adjusted Outcome Measures From Cohort Studies

Eight cohort studies calculated aOR for baseline characteristics between conservative management and either pharmacological therapy, ligation, or pharmacological therapy followed by ligation (7, 26, 28, 30, 31, 33–35). Table 10 shows a statistically significant higher risk for mortality and an overall lower risk for morbidity, especially BPD, in the conservatively managed group.

**Table 10:** Adjusted odds ratio for outcome parameters in non-randomized cohort studies.

**Comparison**	**References**	**Adjusted for**	**Mortality**	**BPD**	**NEC**	**IVH**	**ROP**
Conservative management vs. pharmacological treatment	Härkin et al. (26)	GA, SGA, RDS, delivery hospital, and ACS	**2.0 (1.1–3.3)**	**0.5 (0.3–0.9)**	0.8 (0.6–1.1)	0.9 (0.6–1.4)	0.9 (0.4–2.0)
	Laughon et al. (28)	BW, GA, inborn status, and ACS	**1.7 (1.4–2.0)**	**0.7 (0.6–0.8)**	NS	NS	**0.7 (0.6–0.8)**
	Mohamed et al. (31)	GA, BW, sex, and maternal conditions	**0.51 (0.25–0.99)**	0.71 (0.28–1.80)	0.84 (0.46–1.53)	1.31 (0.61–2.81)	1.31 (0.51–3.38)
Conservative management vs. ligation	Härkin et al. (26)	GA, SGA, RDS, delivery hospital, and ACS	1.0 (0.6–2.0)	**0.3 (0.1–0.5)**	**0.5 (0.3–0.8)**	**0.2 (0.1–0.4)**	1.7 (0.3–10.0)
	Sung et al. (34)	GA, BW, SGA, sex, AS^5^, and ACS	0.8^§^ (0.3–2.2)	**0.4**^**§**^ **(0.2–0.8)**	–	–	–
Conservative management vs. pharmacological treatment and/or ligation	Härkin et al. (26)	GA, SGA, RDS, delivery hospital, and ACS	**3.3 (1.4–5.0)**	**0.2 (0.1–0.3)**	0.7 (0.4–1.1)	0.8 (0.5–1.3)	0.7 (0.3–1.7)

*Data presented as adjusted odds ratio (95% confidence interval) after multivariable logistic regression unless otherwise specified. Significant differences are bold*.

§ *Propensity score adjusted*.

*ACS; antenatal corticosteroids, AS^5^; Apgar score at 5 min postpartum, BPD; bronchopulmonary dysplasia, BW; birth weight, CS; Cesarean section, GA; gestational age, IVH; intraventricular hemorrhage, NEC; necrotizing enterocolitis, NS; not significant, RDS; respiratory distress syndrome, ROP; retinopathy of prematurity, SGA; small for gestational age*.

#### Adjusted Composite Outcome Measures From Cohort Studies

Studies that calculated an adjusted composite outcome, mainly involving mortality and/or BPD, observed lower aOR after conservative management in comparison with pharmacological treatment (30), to ligation alone (7, 30, 34), and pharmacological therapy and/or ligation (7, 30) (Table 11). One study defined composite outcome as survival without death or BPD and found no difference between conservative treatment and pharmacological treatment (aOR, 1.72 [0.92–3.23]) (31).

**Table 11:** Adjusted odds ratio for composite outcome in cohort studies.

			**Composite outcome according to comparison**		
**References**	**Adjusted for**	**Cohort**	**Conservative management vs. pharmacological treatment**	**Conservative management vs. ligation**	**Conservative management vs. pharmacological treatment and/or ligation**
Lokku et al. (30)^†^	GA, SGA, sex, SNAP II score >20, outborn, maternal hypertension, and ACS	2006–2008 2009–2012	1.05 (0.79–1.41) **0.61 (0.51–0.74)**	**0.53 (0.31–0.93)** **0.24 (0.13–0.43)**	**0.36 (0.23–0.56)** **0.27 (0.19–0.38)**
Mirea et al. (7)^†^	GA, SGA, sex, SNAP II >20, year of birth, site, inborn/outborn,		0.91 (0.72–1.16)	**0.56 (0.37–0.87)**	**0.43 (0.30–0.60)**
	maternal hypertension, gestational		0.99^§^ (0.76–1.28)	**0.52**^**§**^ **(0.29–0.93)**	**0.45**^**§**^ **(0.29–0.70)**
	diabetes, chorioamnionitis, and ACS		0.99^§§^ (0.72–1.35)	**0.59**^**§§**^ **(0.42–0.82)**	**0.80**^**§§**^ **(0.66–0.97)**
Sung et al. (34)^††^	GA, BW, SGA, AS^5^, sex, and ACS		–	**0.5**^**§**^ **(0.2–0.9)**	–

*Data presented as adjusted odds ratio (95% confidence interval) after multivariable logistic regression unless otherwise specified. Significant differences are bold*.

*ACS; antenatal corticosteroids, AS^5^; Apgar score at 5 min postpartum, BW; birth weight, GA; gestational age, SGA; small for gestational age, SNAP II; Score for Neonatal Acute Physiology, version II*.

§ *Propensity score adjusted*.

§§ *Propensity score matched*.

*Composite outcome defined as mortality or any severe morbidity (IVH grade ≥3, or periventricular leukomalacia, ROP stage ≥3, BPD defined as oxygen need at 36 weeks postmenstrual age and NEC stages ≥2)*.

*Composite outcome defined as mortality or BPD*.

## Discussion

In this systematic review, we reviewed the available literature of the last two decades regarding a conservative management for a PDA in preterm infants. Meta-analysis of the included RCTs showed no differences in outcome for the conservative management group compared with active treatment groups. This is in line with a recent network meta-analysis that demonstrated no differences in severe neonatal morbidities between pharmacological treatment and no (active) treatment (4). This meta-analysis also included RCTs with an overall high rate of open-label active treatment in the conservative management (no treatment/placebo) group. However, our meta-analysis only including strict conservative management regimens RCTs, also showed no differences in mortality and/or morbidity in the small number of patients included.

Contrarily, meta-analysis of the cohort studies suggest an association with a significant higher risk for mortality in the conservative management group compared with most active treatment groups. Our meta-analysis hereby adds to the available evidence indicating an association between PDA and mortality (2); however, causality remains unproven. Remarkably, a significant lower risk for severe neonatal morbidities was found in our meta-analysis of the cohort studies in the conservative management group compared with various active treatment regimens.

The risk of bias of the included cohort studies was classified as moderate. The main risk of bias was treatment selection bias or confounding by indication, since patients could be managed conservatively due to contraindications for ibuprofen/indomethacin or because of a non-hsPDA. The lower incidence of neonatal morbidity might be due to survival bias, as patients who died cannot develop BPD. Furthermore, patients at the highest risk to develop (severe) morbidities are more likely to die. This might be enhanced by the exclusion of early neonatal death in four cohort studies (7, 30, 32, 34). Many cohort studies were derived from databases (7, 26, 30–34), which are at risk of poor diagnostic precision. These biases might explain the observed higher risk for mortality on the one hand, and the lower risk for morbidity on the other hand for conservatively managed patients compared with active treatment regimens.

Our subgroup analysis including patients without a PDA (“best-case scenario”) further suggests treatment selection bias, as patients with the highest *a priori* risk for mortality were possibly not treated for their PDA while low-risk patients might have been excluded from retrospective cohort studies. The higher risk for mortality lost significance, while the lower risk for morbidity was even more pronounced. This supports our hypothesis that the decreased risk for morbidity might be due to the inclusion of relatively well children in the conservative treatment group. Furthermore, treated patients were systematically younger and/or smaller than conservatively treated patients. We also included a subgroup analysis of patients with an echocardiographic confirmed PDA (“worst-case scenario”) in an attempt to exclude preterm infants with a small PDA that did not necessitated treatment as it would close spontaneously. In this subgroup, only the risk for BPD was significantly lower for the conservative management group. This might be due to the clinicians' tendency to treat a PDA, even with the same echocardiographic PDA characteristics, in case of ventilator dependency which in itself is a risk factor for BPD (36).

The higher risk for mortality and lower risk for morbidity in conservatively managed infants remained significant in three cohort studies after adjustment for baseline characteristics (26, 28, 34). Only one study observed a significantly lower risk for mortality for conservatively managed infants, without a difference in risk for morbidity. This might be due to differences in neonatal practice overall, since they compared a first epoch characterized by active pharmacological treatment (2001–2009) with a second epoch with predominantly a conservative management (2010–2014) (31). The composite outcome, heterogeneously defined as mortality and/or morbidity, was significantly lower in the conservatively managed group (7, 30, 34).

Adjustment for baseline perinatal characteristics does not completely reduce treatment selection bias in the cohort studies, since they cannot correct for all relevant clinical conditions after birth and potential unmeasured confounders. These confounders might have influenced the clinician's decision whether or not to treat a PDA in an infant. The importance of these confounders might be crucial, since in analogy the association between ligation and morbidity lost significance only after the adjustment for postnatal, preligation covariates like sepsis, cardiovascular drug support, NEC, and severe IVH (9).

We could not replicate the finding that prophylactic treatment significantly reduces the risk of IVH (3). This might be due to our exclusion criteria, since most trials regarding prophylactic indomethacin were conducted before 2000 and/or had >25% open-label active treatment in the placebo group (37). In the only included cohort comparing conservative management to prophylactic treatment, although conservatively treated infants were significantly less mature, there was no difference in IVH in both the adjusted and unadjusted analysis (28).

This systematic literature review highlights the main pitfalls of the available evidence regarding PDA treatment in preterm and/or VLBW infants. Eligible RCTs are scarce, due to our strict inclusion criteria. Consequentially, most included studies were retrospective cohort studies with the accompanying heterogeneity and higher risk of bias. Heterogeneity occurred due to different diagnostic approaches and variety in used definition of (hs)PDA. Conservative management in the included studies was predominantly classified as no treatment with indomethacin, ibuprofen, acetaminophen, and/or ligation. If specifically defined, it was highly variable from watchful waiting to the use of diuretics and/or fluid restriction and/or ventilator adjustments.

With the currently available literature regarding conservative PDA management, one might conclude that it appears safe to wait for delayed spontaneous closure based on RCT data. However, cohort studies suggest that conservative management is associated with a higher risk for mortality, but a lower risk for morbidity, albeit with a very low level of evidence. Therefore, a conservative management cannot be generalized to all preterm infants with a PDA and considered evidence-based practice at this moment.

Instead of dichotomizing a PDA as present or not, one should consider the PDA as a spectrum in which the amount of shunt volume across the PDA is thought to be associated with adverse outcome. To asses shunt volume (neonatologist performed), echocardiography could play an important role (38). Additional objective measurements indicative of transductal left-to-right shunt volume, rather than DA diameter alone, could better indicate hemodynamic significance, for example, the PDA severity score (39).

The high amount of active treatment in cohort studies and open-label treatment in RCTs suggests that in case of PDA associated morbidities clinicians might try to rule out a putative causal role of a PDA and therefor initiate active treatment in an attempt to achieve PDA closure. As included cohort studies mainly stratified patients regarding their final PDA treatment (“as treated”), instead of the initial management to which RCTs randomize (“intention to treat”), our meta-analysis could not correct for treatment selection bias, which is one of the main limitations.

The tendency of clinicians to actively close the DA in case of associated findings, hence absence of clinical equipoise, remains one of the main limitations in RCTs. In the PDA TOLERATE trial (40), 48% of the patients allocated to conservative management received open-label active treatment, referred to as “rescue” treatment (40). For future RCTs, we would suggest defining “open-label treatment” criteria as “rescue treatment” insinuates treatment is superior to conservative management in preterm infants with a PDA for which evidence is lacking. Together with the different types of bias in both RCTs and cohort studies rescue treatment contributes to the everlasting conundrum on PDA management.

In conclusion, we found no differences in outcome in the included RCTs. Our meta-analysis highlights the lack of high-quality evidence for conservative management for PDA in preterm infants.

The current trend toward conservative management cannot be justified based on these scarce, mainly retrospective and very heterogeneous cohort studies. Further cohort studies will not be able to provide a final and conclusive answer to the question whether we should consider a PDA in preterm infants as an epiphenomenon which can be managed conservatively or as an important causal factor or contributing factor to adverse outcome in preterm infants. High-quality RCTs with a conservative management group with a limited—preferably without—open-label treatment rate are needed to elucidate the conundrum whether or not to treat a PDA in extremely preterm infants.

## Data Availability Statement

The original contributions presented in the study are included in the article/Supplementary Material, further inquiries can be directed to the corresponding author/s.

## Author Contributions

TH, EJ, and WB initiated the idea for this systematic review and meta-analysis. TH, WB, and WO contributed to the design of the study. Data acquisition and analysis were done by TH and EJ. WO, EK, PA, and WB revised the article critically for important intellectual content. All authors approved the final version.

## Conflict of Interest

The authors declare that the research was conducted in the absence of any commercial or financial relationships that could be construed as a potential conflict of interest.
