# Supplementary material for: Conservative Management of Patent Ductus Arteriosus in Preterm Infants—A Systematic Review and Meta-Analyses Assessing Differences in Outcome Measures Between Randomized Controlled Trials and Cohort Studies
Source: Front Pediatr. 2021 Feb 25;9:626261. doi: 10.3389/fped.2021.626261 (PMC7946967; doi:10.3389/fped.2021.626261)
Supplement: Supplementary file 1 [file Data_Sheet_1.docx]

Supplementary Material

**Supplement 1 –** Forrest plots of randomized controlled trials

**Supplement 2 –** GRADE Randomized controlled trials

**Supplement 3 –** Forrest plots of cohort studies

**Supplement 4 –** GRADE Cohort studies

**Supplement 5 –** Forrest plots of cohort studies including patients without PDA

**Supplement 1 –** Forrest plots of randomized controlled trials

a)


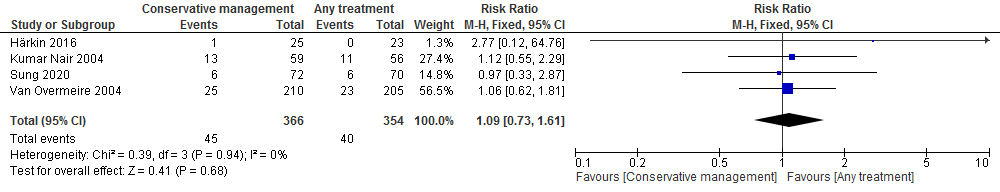


b)


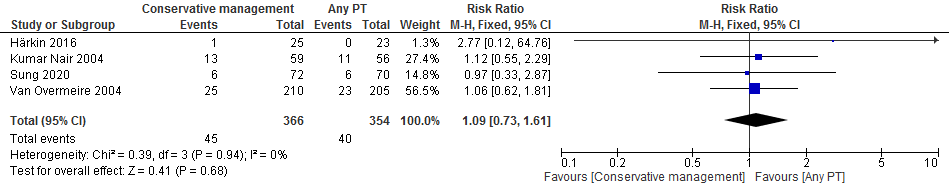


c)

**
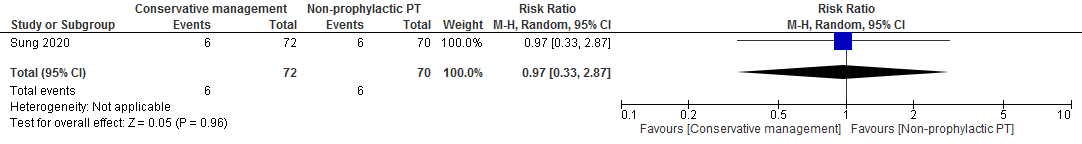
**

d)

**
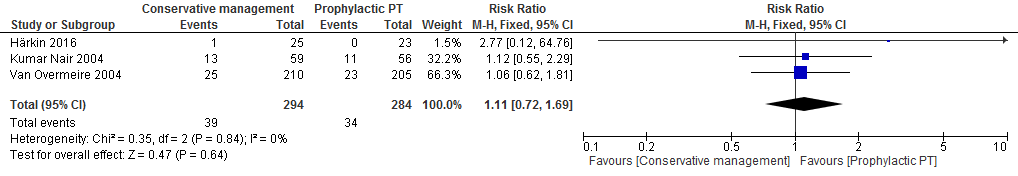
**

**Figure S1-1 –** Conservative management versus a) any treatment; b) any pharmacological treatment; c) non-prophylactic pharmacological treatment, and; d) prophylactic pharmacological treatment for patent ductus arteriosus in RCTs – Mortality

a)


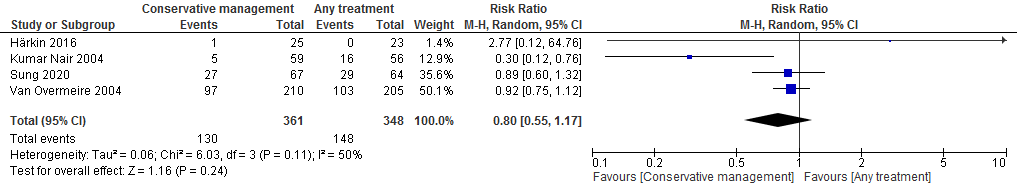


b)


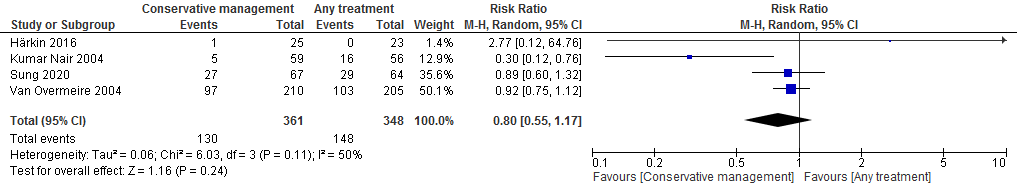


c)


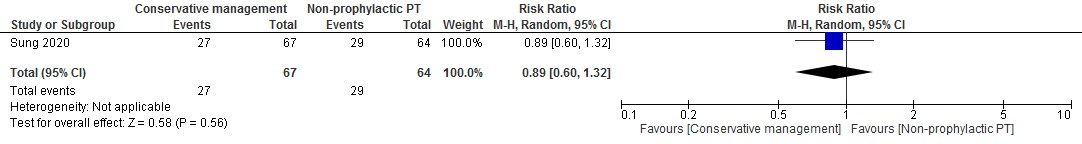


d)


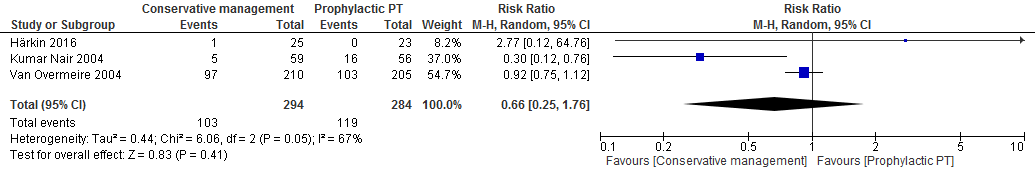


**Figure S1-2 –** Conservative management versus a) any treatment; b) any pharmacological treatment; c) non-prophylactic pharmacological treatment, and; d) prophylactic pharmacological treatment for patent ductus arteriosus in RCTs – BPD (any definition)

a)

**
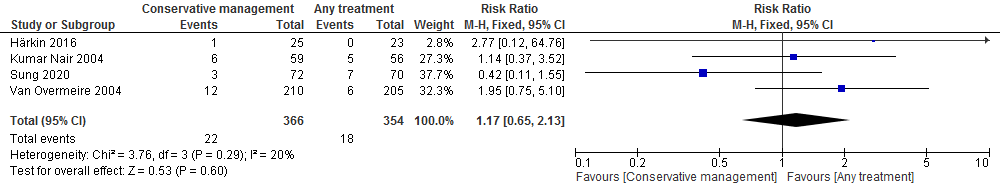
**

b)

**
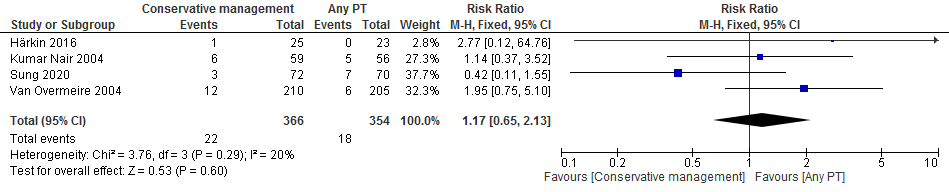
**

c)

**
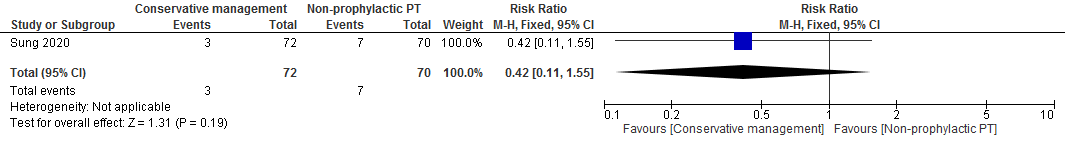
**

d)

**
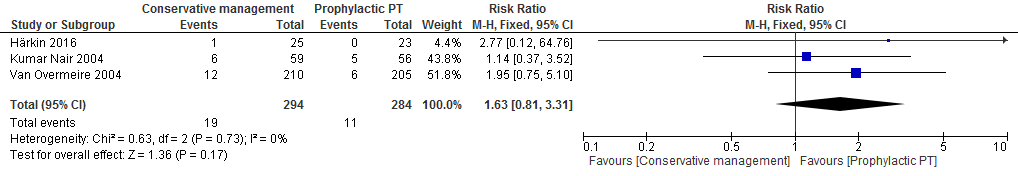
**

**Figure S1-3 –** Conservative management versus a) any treatment; b) any pharmacological treatment; c) non-prophylactic pharmacological treatment, and; d) prophylactic pharmacological treatment for patent ductus arteriosus in RCTs – NEC (any stage)

a)

**
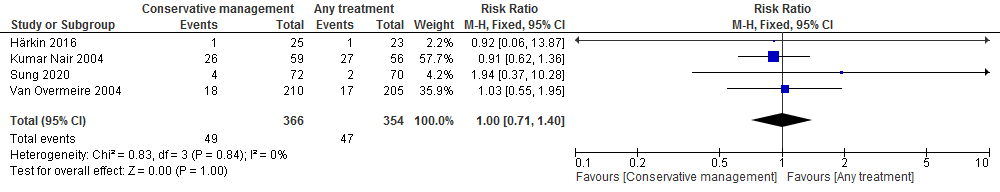
**

b)

**
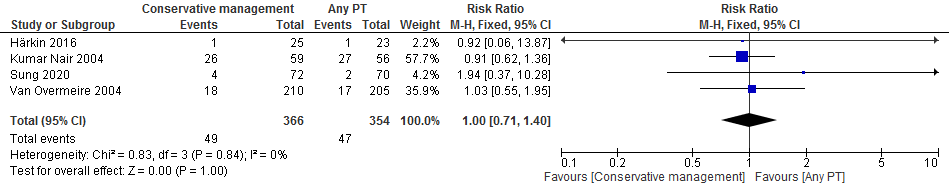
**

c)

**
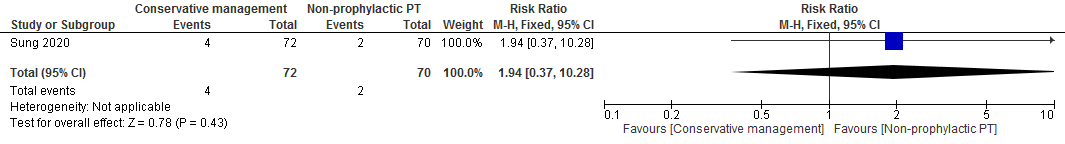
**

d)

**
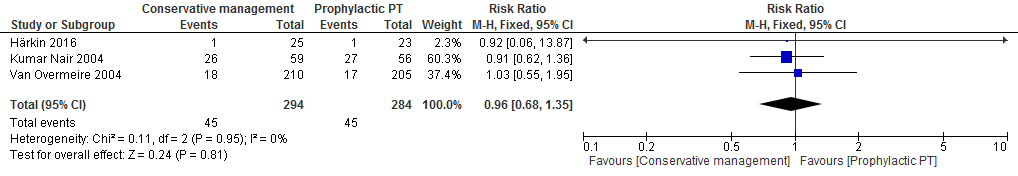
**

**Figure S1-4 –** Conservative management versus a) any treatment; b) any pharmacological treatment; c) non-prophylactic pharmacological treatment, and; d) prophylactic pharmacological treatment for patent ductus arteriosus in RCTs – IVH (any grade)

a)

**
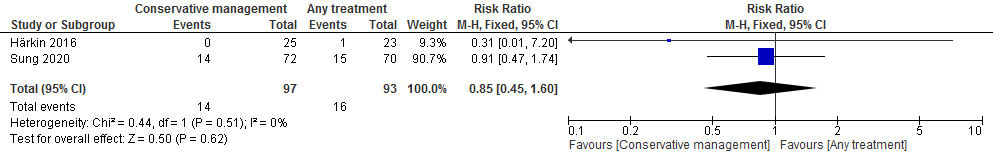
**

b)


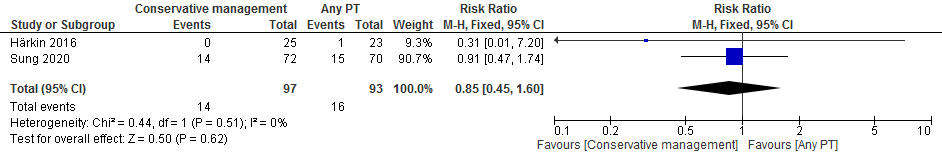


c)


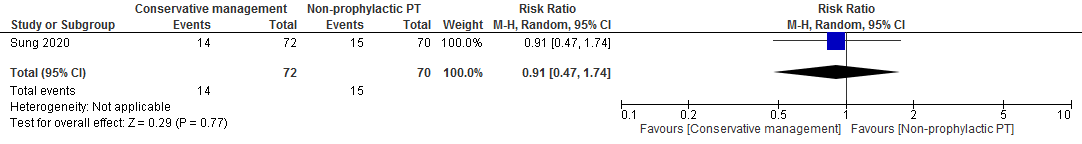


d)


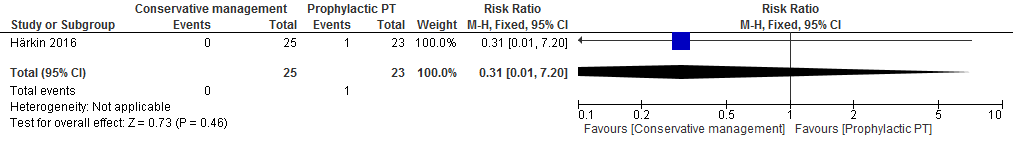


**Figure S1-5 –** Conservative management versus a) any treatment; b) any pharmacological treatment; c) non-prophylactic pharmacological treatment, and; d) prophylactic pharmacological treatment for patent ductus arteriosus in RCTs – ROP (any stage)

**Supplement 2 –** GRADE Randomized controlled trials

**Author(s)**: T. Hundscheid, E.J.S. Jansen, W. Onland, E.M.W. Kooi, P. Andriessen, W.P. de Boode

**Question**: Conservative management compared to any active treatment for patent ductus arteriosus in preterm and/or very low birth weight infants

| **Certainty assessment** | | | | | | | **№ of patients** | | **Effect** | | **Certainty** | **Importance** |
| --- | --- | --- | --- | --- | --- | --- | --- | --- | --- | --- | --- | --- |
| **№ of studies** | **Study design** | **Risk of bias** | **Inconsistency** | **Indirectness** | **Imprecision** | **Other considerations** | **conservative management** | **any active treatment** | **Relative (95% CI)** | **Absolute (95% CI)** |  |  |
| **Mortality** | | | | | | | | | | | | |
| 4 | randomised trials | not serious | not serious | not serious | serious ^a,b^ | none | 45/366 (12.3%) | 40/354 (11.3%) | **RR 1.09** (0.73 to 1.61) | **20 fewer per 1.000** (from 70 fewer to 30 more) | ⨁⨁⨁◯ MODERATE | CRITICAL |
| **BPD (any definition)** | | | | | | | | | | | | |
| 4 | randomised trials | not serious | serious ^c,d^ | not serious | serious ^a,b^ | none | 130/361 (36.0%) | 148/348 (42.5%) | **RR 0.80** (0.55 to 1.17) | **60 more per 1.000** (from 70 fewer to 190 more) | ⨁⨁◯◯ LOW | IMPORTANT |
| **NEC (any stage)** | | | | | | | | | | | | |
| 4 | randomised trials | not serious | not serious | not serious | serious ^a,b^ | none | 22/366 (6.0%) | 18/354 (5.1%) | **RR 1.17** (0.65 to 2.13) | **20 fewer per 1.000** (from 100 fewer to 60 more) | ⨁⨁⨁◯ MODERATE | IMPORTANT |
| **IVH (any grade)** | | | | | | | | | | | | |
| 4 | randomised trials | not serious | not serious | not serious | serious ^a,b^ | none | 49/366 (13.4%) | 47/354 (13.3%) | **RR 1.00** (0.71 to 1.40) | **10 more per 1.000** (from 50 fewer to 60 more) | ⨁⨁⨁◯ MODERATE | IMPORTANT |
| **ROP (any stage)** | | | | | | | | | | | | |
| 2 | randomised trials | not serious | not serious | not serious | serious ^a,b^ | none | 14/72 (19.4%) | 16/93 (17.2%) | **RR 0.85** (0.45 to 1.60) | **40 more per 1.000** (from 70 fewer to 150 more) | ⨁⨁⨁◯ MODERATE | IMPORTANT |

CI: Confidence interval; RR: Risk ratio

*Explanations*

a. Number of events <300
b. Confidence interval width >0.25
c. Statistical test for heterogeneity shows a P value <0.05
d. The I^2^ is >50%

**Author(s)**: T. Hundscheid, E.J.S. Jansen, W. Onland, E.M.W. Kooi, P. Andriessen, W.P. de Boode

**Question**: Conservative management compared to any pharmacological treatment for patent ductus arteriosus in preterm and/or very low birth weight infants

| **Certainty assessment** | | | | | | | **№ of patients** | | **Effect** | | **Certainty** | **Importance** |
| --- | --- | --- | --- | --- | --- | --- | --- | --- | --- | --- | --- | --- |
| **№ of studies** | **Study design** | **Risk of bias** | **Inconsistency** | **Indirectness** | **Imprecision** | **Other considerations** | **conservative management** | **any pharmacological treatment** | **Relative (95% CI)** | **Absolute (95% CI)** |  |  |
| **Mortality** | | | | | | | | | | | | |
| 4 | randomised trials | not serious | not serious | not serious | serious ^a,b^ | none | 45/366 (12.3%) | 40/354 (11.3%) | **RR 1.09** (0.73 to 1.61) | **20 fewer per 1.000** (from 70 fewer to 30 more) | ⨁⨁⨁◯ MODERATE | CRITICAL |
| **BPD (any definition)** | | | | | | | | | | | | |
| 4 | randomised trials | not serious | serious ^c,d^ | not serious | serious ^a,b^ | none | 130/361 (36.0%) | 148/348 (42.5%) | **RR 0.80** (0.55 to 1.17) | **60 more per 1.000** (from 70 fewer to 190 more) | ⨁⨁◯◯ LOW | IMPORTANT |
| **NEC (any stage)** | | | | | | | | | | | | |
| 4 | randomised trials | not serious | not serious | not serious | serious ^a,b^ | none | 22/366 (6.0%) | 18/354 (5.1%) | **RR 1.17** (0.65 to 2.13) | **30 fewer per 1.000** (from 100 fewer to 50 more) | ⨁⨁⨁◯ MODERATE | IMPORTANT |
| **IVH (any grade)** | | | | | | | | | | | | |
| 4 | randomised trials | not serious | not serious | not serious | serious ^a,b^ | none | 49/366 (13.4%) | 47/354 (13.3%) | **RR 1.00** (0.71 to 1.40) | **10 more per 1.000** (from 50 fewer to 60 more) | ⨁⨁⨁◯ MODERATE | IMPORTANT |
| **ROP (any stage)** | | | | | | | | | | | | |
| 2 | randomised trials | not serious | not serious | not serious | serious ^a,b^ | none | 14/72 (19.4%) | 16/93 (17.2%) | **RR 0.85** (0.45 to 1.60) | **40 more per 1.000** (from 70 fewer to 150 more) | ⨁⨁⨁◯ MODERATE | IMPORTANT |

CI: Confidence interval; RR: Risk ratio

*Explanations*

a. Number of events <300
b. Confidence interval width >0.25
c. Statistical test for heterogeneity shows a P value <0.05
d. The I^2^ is >50%

**Author(s)**: T. Hundscheid, E.J.S. Jansen, W. Onland, E.M.W. Kooi, P. Andriessen, W.P. de Boode

**Question**: Conservative management compared to non-prophylactic pharmacological treatment for patent ductus arteriosus in preterm and/or very low birth weight infants

| **Certainty assessment** | | | | | | | **№ of patients** | | **Effect** | | **Certainty** | **Importance** |
| --- | --- | --- | --- | --- | --- | --- | --- | --- | --- | --- | --- | --- |
| **№ of studies** | **Study design** | **Risk of bias** | **Inconsistency** | **Indirectness** | **Imprecision** | **Other considerations** | **conservative management** | **non-prophylactic pharmacological treatment** | **Relative (95% CI)** | **Absolute (95% CI)** |  |  |
| **Mortality** | | | | | | | | | | | | |
| 1 ^a,b^ | randomised trials | not serious | not serious | not serious | serious ^a,b^ | none | 6/72 (8.3%) | 6/70 (8.6%) | **RR 0.97** (0.33 to 2.87) | **3 fewer per 1.000** (from 57 fewer to 160 more) | ⨁⨁⨁◯ MODERATE |  |
| **BPD (any definition)** | | | | | | | | | | | | |
| 1 | randomised trials | not serious | not serious | not serious | serious ^a,b^ | none | 27/67 (40.3%) | 29/64 (45.3%) | **RR 0.89** (0.60 to 1.32) | **50 more per 1.000** (from 120 fewer to 220 more) | ⨁⨁⨁◯ MODERATE |  |
| **NEC (any stage)** | | | | | | | | | | | | |
| 1 | randomised trials | not serious | not serious | not serious | serious ^a,b^ | none | 3/72 (4.2%) | 7/70 (10.0%) | **RR 0.42** (0.11 to 1.55) | **58 fewer per 1.000** (from 89 fewer to 55 more) | ⨁⨁⨁◯ MODERATE |  |
| **IVH (any grade)** | | | | | | | | | | | | |
| 1 | randomised trials | not serious | not serious | not serious | serious ^a,b^ | none | 4/72 (5.6%) | 2/70 (2.9%) | **RR 1.94** (0.37 to 10.28) | **27 more per 1.000** (from 18 fewer to 265 more) | ⨁⨁⨁◯ MODERATE |  |
| **ROP (any stage)** | | | | | | | | | | | | |
| 1 | randomised trials | not serious | not serious | not serious | serious ^a,b^ | none | 14/72 (19.4%) | 15/70 (21.4%) | **RR 0.91** (0.47 to 1.74) | **19 fewer per 1.000** (from 114 fewer to 159 more) | ⨁⨁⨁◯ MODERATE |  |

CI: Confidence interval; RR: Risk ratio

*Explanations*

a. Number of events <300
b. Confidence interval width >0.25

**Author(s)**: T. Hundscheid, E.J.S. Jansen, W. Onland, E.M.W. Kooi, P. Andriessen, W.P. de Boode

**Question**: Conservative management compared to prophylactic pharmacological treatment for patent ductus arteriosus in preterm and/or very low birth weight infants

| **Certainty assessment** | | | | | | | **№ of patients** | | **Effect** | | **Certainty** | **Importance** |
| --- | --- | --- | --- | --- | --- | --- | --- | --- | --- | --- | --- | --- |
| **№ of studies** | **Study design** | **Risk of bias** | **Inconsistency** | **Indirectness** | **Imprecision** | **Other considerations** | **conservative management** | **prophylactic pharmacological treatment** | **Relative (95% CI)** | **Absolute (95% CI)** |  |  |
| **Mortality** | | | | | | | | | | | | |
| 3 | randomised trials | not serious | not serious | not serious | serious ^a,b^ | none | 39/294 (13.3%) | 34/284 (12.0%) | **RR 1.11** (0.72 to 1.69) | **10 fewer per 1.000** (from 70 fewer to 40 more) | ⨁⨁⨁◯ MODERATE | CRITICAL |
| **BPD (any definition)** | | | | | | | | | | | | |
| 3 | randomised trials | not serious | serious ^c,d^ | not serious | serious ^a,b^ | none | 103/294 (35.0%) | 119/284 (41.9%) | **RR 0.66** (0.25 to 1.76) | **60 more per 1.000** (from 70 fewer to 190 more) | ⨁⨁◯◯ LOW | IMPORTANT |
| **NEC (any stage)** | | | | | | | | | | | | |
| 3 | randomised trials | not serious | not serious | not serious | serious ^a,b^ | none | 19/294 (6.5%) | 11/284 (3.9%) | **RR 1.63** (0.81 to 3.31) | **20 fewer per 1.000** (from 100 fewer to 60 more) | ⨁⨁⨁◯ MODERATE | IMPORTANT |
| **IVH (any grade)** | | | | | | | | | | | | |
| 3 | randomised trials | not serious | not serious | not serious | serious ^a,b^ | none | 45/294 (15.3%) | 45/284 (15.8%) | **RR 0.96** (0.68 to 1.35) | **10 more per 1.000** (from 50 fewer to 60 more) | ⨁⨁⨁◯ MODERATE | IMPORTANT |
| **ROP (any stage)** | | | | | | | | | | | | |
| 1 | randomised trials | not serious | not serious | not serious | serious ^a,b^ | none | 0/25 (0.0%) | 1/23 (4.3%) | **RR 0.31** (0.01 to 7.20) | **40 more per 1.000** (from 70 fewer to 150 more) | ⨁⨁⨁◯ MODERATE | IMPORTANT |

CI: Confidence interval; RR: Risk ratio

*Explanations*

a. Number of events <300
b. Confidence interval width >0.25
c. Statistical test for heterogeneity shows a P value <0.05
d. The I^2^ is >50%

**Supplement 3 –** Forrest plots of cohort studies

a)
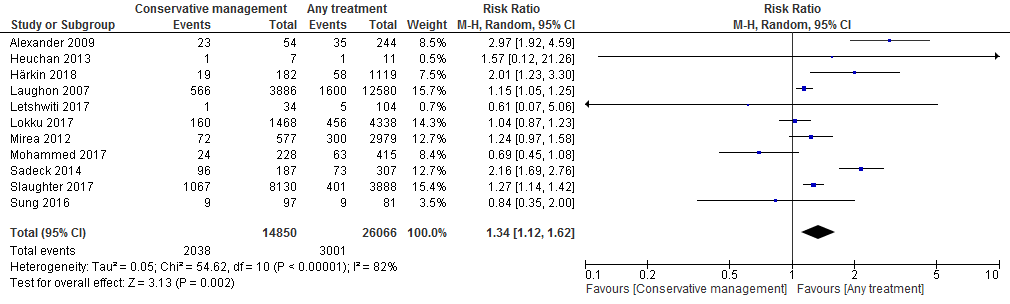


b)


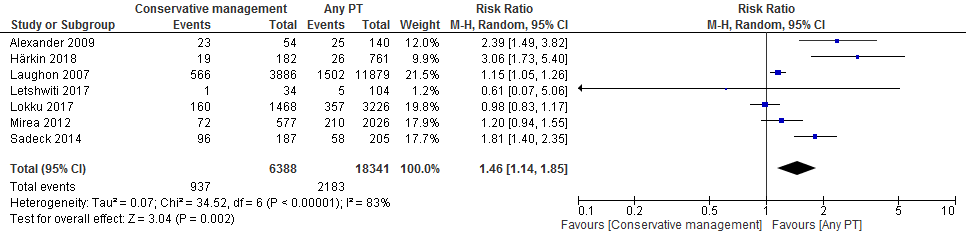


c)
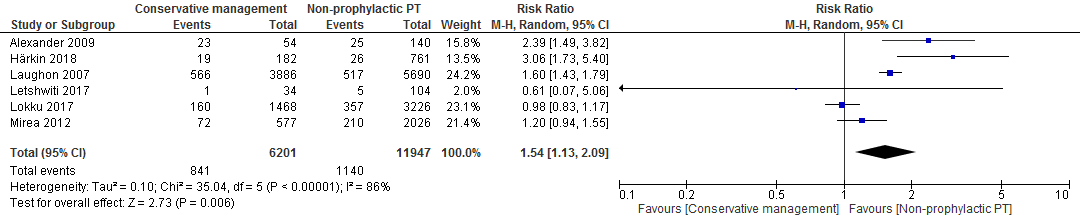


d)
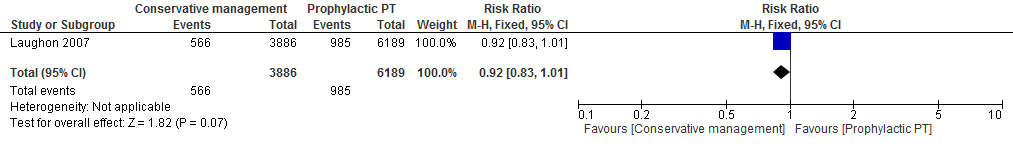


e)

**
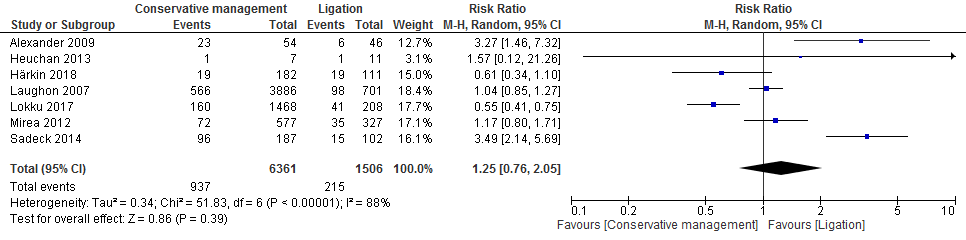
Figure S3-1 –** Conservative management versus a) any treatment; b) any pharmacological treatment; c) non-prophylactic pharmacological treatment; d) prophylactic pharmacological treatment, and; e) ligation for patent ductus arteriosus in cohort studies – Mortality

a)
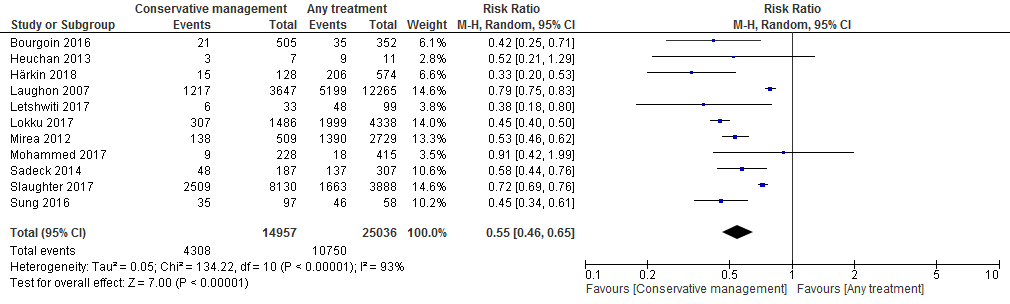


b)
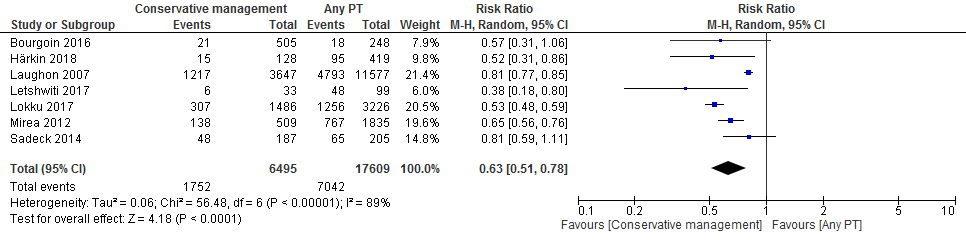


c)


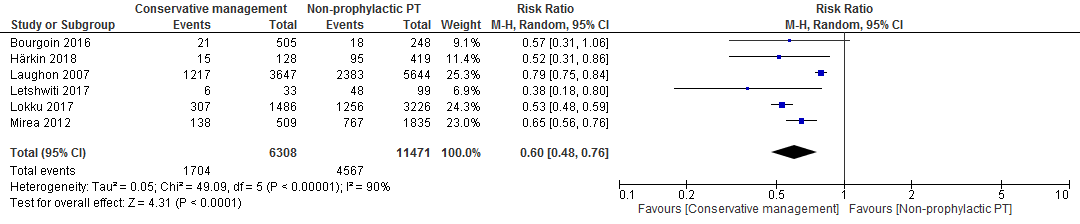


d)


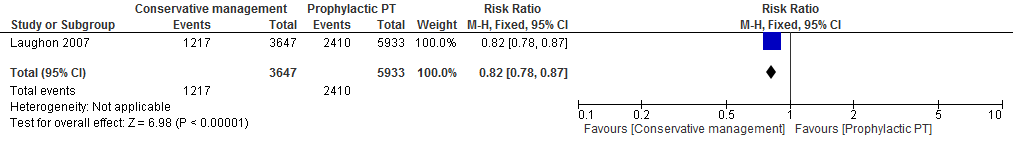


e)

**
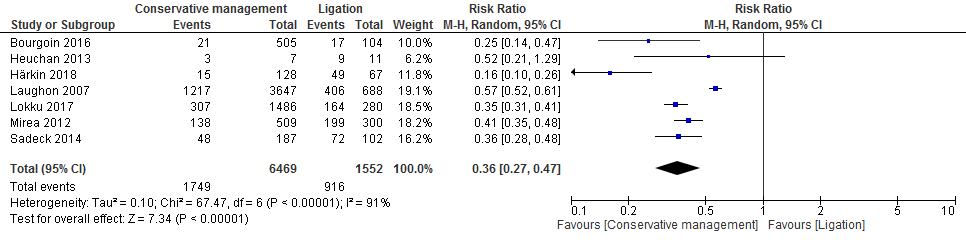
Figure S3-2 –** Conservative management versus a) any treatment; b) any pharmacological treatment; c) non-prophylactic pharmacological treatment; d) prophylactic pharmacological treatment, and; e) ligation for patent ductus arteriosus in cohort studies – BPD (any definition)

a)
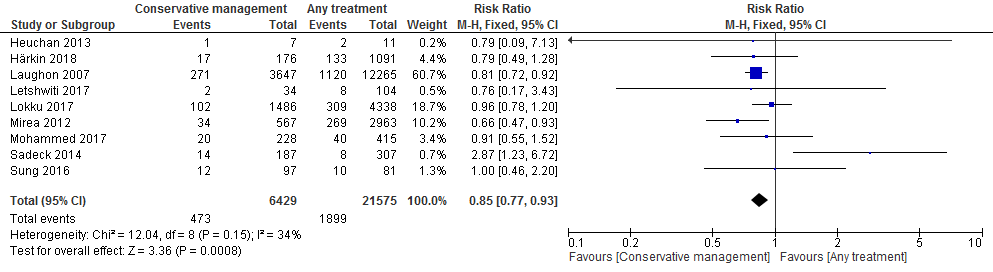


b)
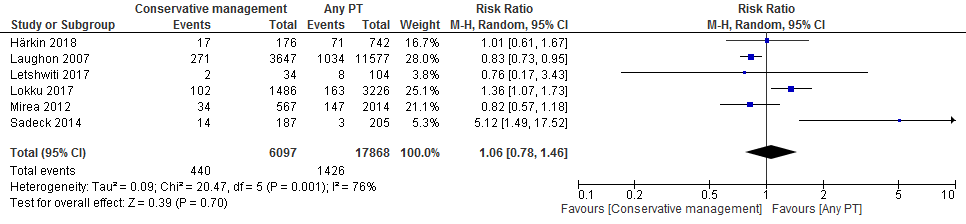


c)
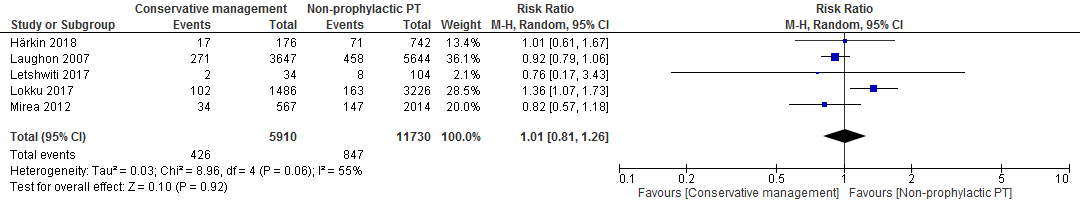


d)
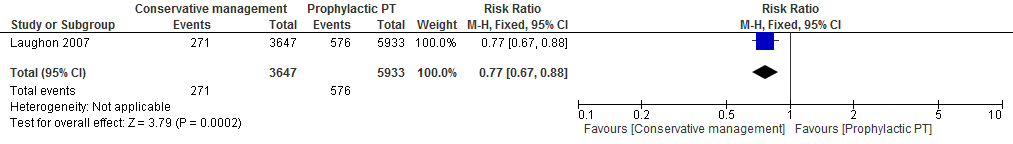


e)
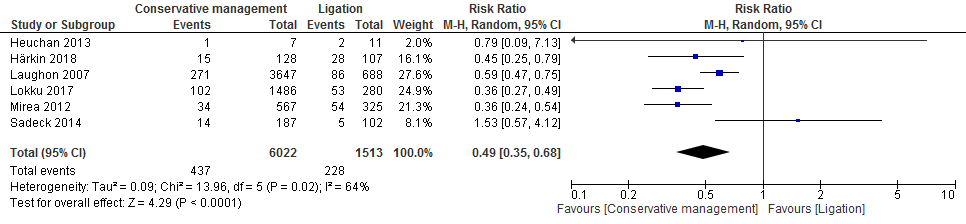


**Figure S3-3 –** Conservative management versus a) any treatment; b) any pharmacological treatment; c) non-prophylactic pharmacological treatment; d) prophylactic pharmacological treatment, and; e) ligation for patent ductus arteriosus in cohort studies – NEC (any stage)

a)
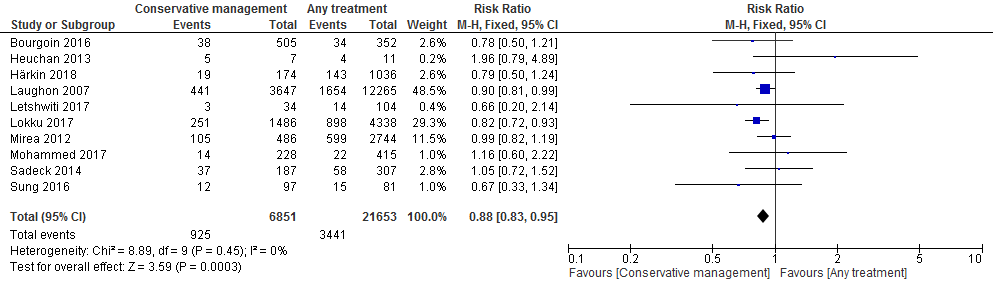


b)
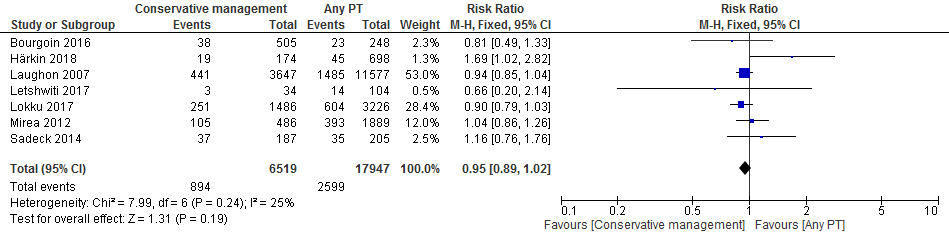


c)
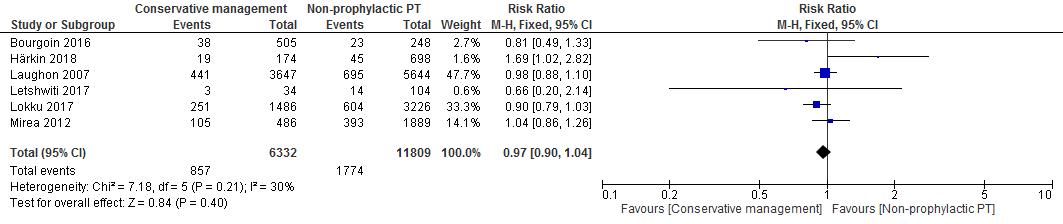


d)
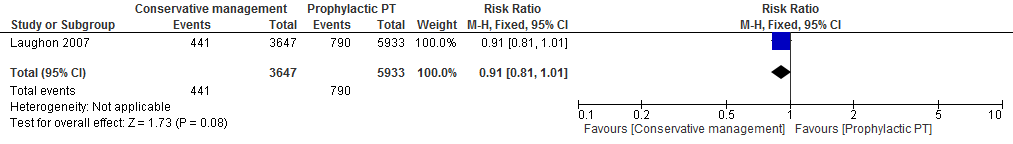


e)
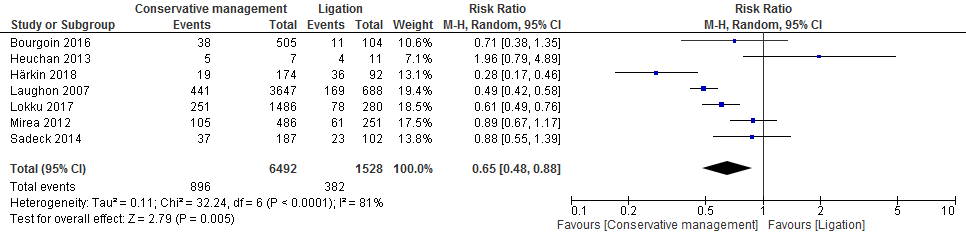


**Figure S3-4 –** Conservative management versus a) any treatment; b) any pharmacological treatment; c) non-prophylactic pharmacological treatment; d) prophylactic pharmacological treatment, and; e) ligation for patent ductus arteriosus in cohort studies – IVH (any grade)

a)
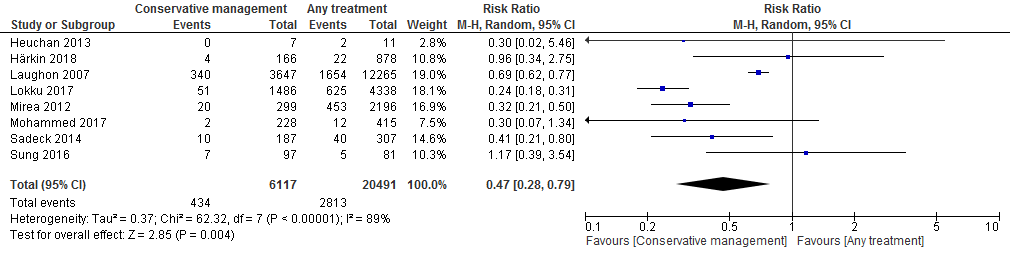


b)
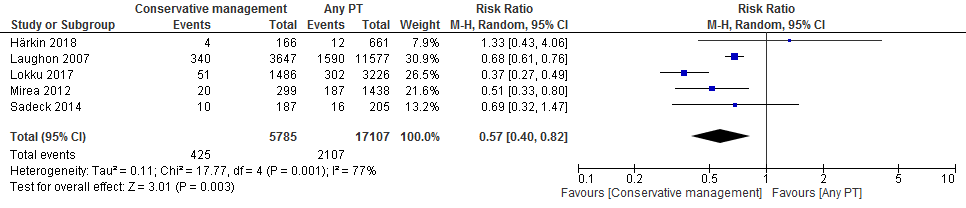


c)
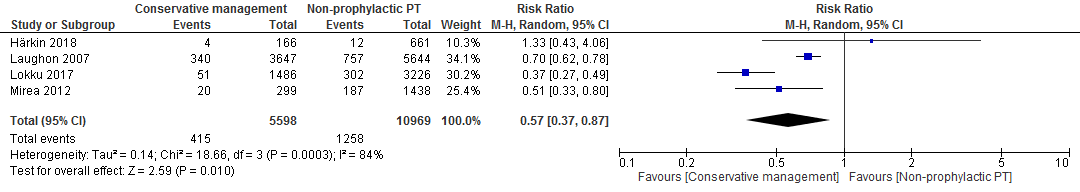


d)
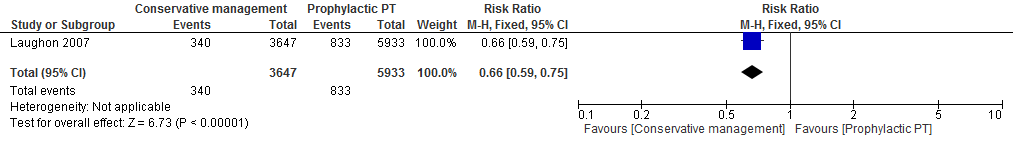


e)
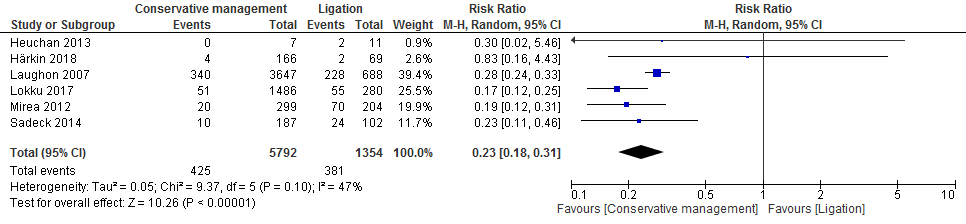


**Figure S3-5 –** Conservative management versus a) any treatment; b) any pharmacological treatment; c) non-prophylactic pharmacological treatment; d) prophylactic pharmacological treatment, and; e) ligation for patent ductus arteriosus in cohort studies – ROP (any stage)

**Supplement 4 –** GRADE Cohort studies

**Author(s)**: T. Hundscheid, E.J.S. Jansen, W. Onland, E.M.W. Kooi, P. Andriessen, W.P. de Boode

**Question**: Conservative management compared to any active treatment for patent ductus arteriosus in preterm and/or very low birth weight infants

| **Certainty assessment** | | | | | | | **№ of patients** | | **Effect** | | **Certainty** | **Importance** |
| --- | --- | --- | --- | --- | --- | --- | --- | --- | --- | --- | --- | --- |
| **№ of studies** | **Study design** | **Risk of bias** | **Inconsistency** | **Indirectness** | **Imprecision** | **Other considerations** | **conservative management** | **any active treatment** | **Relative (95% CI)** | **Absolute (95% CI)** |  |  |
| **Mortality** | | | | | | | | | | | | |
| 11 | observational studies | serious ^a^ | very serious ^b,c^ | not serious | not serious | none | 2038/14850 (13.7%) | 3001/26066 (11.5%) | **RR 1.34** (1.12 to 1.62) | **30 fewer per 1.000** (from 60 fewer to 10 fewer) | ⨁◯◯◯ VERY LOW | CRITICAL |
| **BPD (any definition)** | | | | | | | | | | | | |
| 11 | observational studies | serious ^a^ | very serious ^b,c^ | not serious | not serious | publication bias strongly suspected ^d^ | 4308/14957 (28.8%) | 10750/25036 (42.9%) | **RR 0.55** (0.46 to 0.65) | **180 more per 1.000** (from 120 more to 240 more) | ⨁◯◯◯ VERY LOW | IMPORTANT |
| **NEC (any stage)** | | | | | | | | | | | | |
| 9 | observational studies | serious ^a^ | not serious | not serious | not serious | none | 473/6429 (7.4%) | 1899/21575 (8.8%) | **RR 0.85** (0.77 to 0.93) | **10 more per 1.000** (from 10 more to 20 more) | ⨁◯◯◯ VERY LOW |  |
| **IVH (any grade)** | | | | | | | | | | | | |
| 10 | observational studies | serious ^a^ | not serious | very serious ^e^ | not serious | none | 925/6851 (13.5%) | 3441/21653 (15.9%) | **RR 0.88** (0.83 to 0.95) | **20 more per 1.000** (from 10 more to 30 more) | ⨁◯◯◯ VERY LOW |  |
| **ROP (any stage)** | | | | | | | | | | | | |
| 8 | observational studies | serious ^a^ | very serious ^b,c^ | not serious | not serious | none | 434/6117 (7.1%) | 2813/20491 (13.7%) | **RR 0.47** (0.28 to 0.79) | **60 more per 1.000** (from 20 more to 100 more) | ⨁◯◯◯ VERY LOW |  |

CI: Confidence interval; RR: Risk ratio

*Explanations*

a. Failure to adequately control confounders
b. Statistical test for heterogeneity shows a P value <0.05
c. The I^2^ is >50%
d. Asymmetry of funnel plots
e. Presence of IVH not assessed prior to treatment allocation

**Author(s)**: T. Hundscheid, E.J.S. Jansen, W. Onland, E.M.W. Kooi, P. Andriessen, W.P. de Boode

**Question**: Conservative management compared to any pharmacological treatment for patent ductus arteriosus in preterm and/or very low birth weight infants

| **Certainty assessment** | | | | | | | **№ of patients** | | **Effect** | | **Certainty** | **Importance** |
| --- | --- | --- | --- | --- | --- | --- | --- | --- | --- | --- | --- | --- |
| **№ of studies** | **Study design** | **Risk of bias** | **Inconsistency** | **Indirectness** | **Imprecision** | **Other considerations** | **conservative management** | **any pharmacological treatment** | **Relative (95% CI)** | **Absolute (95% CI)** |  |  |
| **Mortality** | | | | | | | | | | | | |
| 7 | observational studies | serious ^a^ | very serious ^b,c^ | not serious | not serious | none | 937/6388 (14.7%) | 2183/18341 (11.9%) | **RR 1.46** (1.14 to 1.85) | **50 fewer per 1.000** (from 80 fewer to 10 fewer) | ⨁◯◯◯ VERY LOW |  |
| **BPD (any definition)** | | | | | | | | | | | | |
| 7 | observational studies | serious ^a^ | very serious ^b,c^ | not serious | not serious | none | 1752/6495 (27.0%) | 7042/17609 (40.0%) | **RR 0.63** (0.51 to 0.78) | **120 more per 1.000** (from 60 more to 170 more) | ⨁◯◯◯ VERY LOW |  |
| **NEC (any stage)** | | | | | | | | | | | | |
| 6 | observational studies | serious ^a^ | very serious ^b,c^ | not serious | not serious | none | 440/6097 (7.2%) | 1426/17868 (8.0%) | **RR 1.06** (0.78 to 1.46) | **10 fewer per 1.000** (from 30 fewer to 20 more) | ⨁◯◯◯ VERY LOW |  |
| **IVH (any grade)** | | | | | | | | | | | | |
| 7 | observational studies | serious ^a^ | not serious | not serious | not serious | none | 894/6519 (13.7%) | 2599/17947 (14.5%) | **RR 0.95** (0.89 to 1.02) | **10 more per 1.000** (from 0 fewer to 20 more) | ⨁◯◯◯ VERY LOW |  |
| **ROP (any stage)** | | | | | | | | | | | | |
| 5 | observational studies | serious ^a^ | very serious ^b,c^ | not serious | not serious | none | 425/5785 (7.3%) | 2107/17107 (12.3%) | **RR 0.57** (0.40 to 0.82) | **40 more per 1.000** (from 20 more to 60 more) | ⨁◯◯◯ VERY LOW |  |

CI: Confidence interval; RR: Risk ratio

*Explanations*

a. Failure to adequately control confounding
b. Statistical test for heterogeneity shows a P value <0.05
c. The I^2^ is >50%

**Author(s)**: T. Hundscheid, E.J.S. Jansen, W. Onland, E.M.W. Kooi, P. Andriessen, W.P. de Boode

**Question**: Conservative management compared to non-prophylactic pharmacological treatment for patent ductus arteriosus in preterm and/or very low birth weight infants

| **Certainty assessment** | | | | | | | **№ of patients** | | **Effect** | | **Certainty** | **Importance** |
| --- | --- | --- | --- | --- | --- | --- | --- | --- | --- | --- | --- | --- |
| **№ of studies** | **Study design** | **Risk of bias** | **Inconsistency** | **Indirectness** | **Imprecision** | **Other considerations** | **conservative management** | **non-prophylactic pharmacological treatment** | **Relative (95% CI)** | **Absolute (95% CI)** |  |  |
| **Mortality** | | | | | | | | | | | | |
| 6 | observational studies | serious ^a^ | very serious ^b,c^ | not serious | not serious | none | 841/6201 (13.6%) | 1140/11947 (9.5%) | **RR 1.54** (1.13 to 2.09) | **40 fewer per 1.000** (from 70 fewer to 0 fewer) | ⨁◯◯◯ VERY LOW |  |
| **BPD (any definition)** | | | | | | | | | | | | |
| 6 | observational studies | serious ^a^ | very serious ^c,d^ | not serious | not serious | none | 1704/6308 (27.0%) | 4567/11471 (39.8%) | **RR 0.60** (0.48 to 0.76) | **130 more per 1.000** (from 70 more to 180 more) | ⨁◯◯◯ VERY LOW |  |
| **NEC (any stage)** | | | | | | | | | | | | |
| 5 | observational studies | serious ^a^ | very serious ^b,c^ | not serious | not serious | none | 426/5910 (7.2%) | 847/11730 (7.2%) | **RR 1.01** (0.81 to 1.26) | **0 fewer per 1.000** (from 10 fewer to 20 more) | ⨁◯◯◯ VERY LOW |  |
| **IVH (any grade)** | | | | | | | | | | | | |
| 6 | observational studies | serious ^a^ | not serious | not serious | not serious | none | 857/6332 (13.5%) | 1774/11809 (15.0%) | **RR 0.97** (0.90 to 1.04) | **0 fewer per 1.000** (from 10 fewer to 20 more) | ⨁◯◯◯ VERY LOW |  |
| **ROP (any stage)** | | | | | | | | | | | | |
| 4 | observational studies | serious ^a^ | very serious ^c,d^ | not serious | not serious | none | 415/5598 (7.4%) | 1258/10969 (11.5%) | **RR 0.57** (0.37 to 0.87) | **50 more per 1.000** (from 40 more to 60 more) | ⨁◯◯◯ VERY LOW |  |

CI: Confidence interval; RR: Risk ratio

*Explanations*

a. Failure to adequately control confounding
b. Statistical test for heterogeneity shows a P value <0.05
c. The I^2^ is >50%
d. Presence of IVH not assessed prior to treatment allocation

**Author(s)**: T. Hundscheid, E.J.S. Jansen, W. Onland, E.M.W. Kooi, P. Andriessen, W.P. de Boode

**Question**: Conservative management compared to prophylactic pharmacological treatment for patent ductus arteriosus in preterm and/or very low birth weight infants

| **Certainty assessment** | | | | | | | **№ of patients** | | **Effect** | | **Certainty** | **Importance** |
| --- | --- | --- | --- | --- | --- | --- | --- | --- | --- | --- | --- | --- |
| **№ of studies** | **Study design** | **Risk of bias** | **Inconsistency** | **Indirectness** | **Imprecision** | **Other considerations** | **conservative management** | **prophylactic pharmacological treatment** | **Relative (95% CI)** | **Absolute (95% CI)** |  |  |
| **Mortality** | | | | | | | | | | | | |
| 1 | observational studies | serious ^a^ | not serious | not serious | not serious | none | 566/3886 (14.6%) | 985/6189 (15.9%) | **RR 0.92** (0.83 to 1.01) | **10 more per 1.000** (from 0 fewer to 30 more) | ⨁◯◯◯ VERY LOW |  |
| **BPD (any definition)** | | | | | | | | | | | | |
| 1 | observational studies | serious ^a^ | not serious | not serious | not serious | none | 1217/3647 (33.4%) | 2410/5933 (40.6%) | **RR 0.82** (0.78 to 0.87) | **70 more per 1.000** (from 50 more to 90 more) | ⨁◯◯◯ VERY LOW |  |
| **NEC (any stage)** | | | | | | | | | | | | |
| 1 | observational studies | serious ^a^ | not serious | not serious | not serious | none | 271/3647 (7.4%) | 576/5933 (9.7%) | **RR 0.77** (0.67 to 0.88) | **20 more per 1.000** (from 10 more to 30 more) | ⨁◯◯◯ VERY LOW |  |
| **IVH (any grade)** | | | | | | | | | | | | |
| 1 | observational studies | serious ^a^ | not serious | not serious | not serious | none | 441/3647 (12.1%) | 790/5933 (13.3%) | **RR 0.91** (0.81 to 1.01) | **10 more per 1.000** (from 0 fewer to 30 more) | ⨁◯◯◯ VERY LOW |  |
| **ROP (any stage)** | | | | | | | | | | | | |
| 1 | observational studies | serious ^a^ | not serious | not serious | not serious | none | 340/3647 (9.3%) | 833/5933 (14.0%) | **RR 0.66** (0.59 to 0.75) | **50 more per 1.000** (from 30 more to 60 more) | ⨁◯◯◯ VERY LOW |  |

CI: Confidence interval; RR: Risk ratio

*Explanations*

a. Outcome assessment not explicitly described

**Author(s)**: T. Hundscheid, E.J.S. Jansen, W. Onland, E.M.W. Kooi, P. Andriessen, W.P. de Boode

**Question**: Conservative management compared to ligation for patent ductus arteriosus in preterm and/or very low birth weight infants

| **Certainty assessment** | | | | | | | **№ of patients** | | **Effect** | | **Certainty** | **Importance** |
| --- | --- | --- | --- | --- | --- | --- | --- | --- | --- | --- | --- | --- |
| **№ of studies** | **Study design** | **Risk of bias** | **Inconsistency** | **Indirectness** | **Imprecision** | **Other considerations** | **conservative management** | **ligation** | **Relative (95% CI)** | **Absolute (95% CI)** |  |  |
| **Mortality** | | | | | | | | | | | | |
| 7 | observational studies | serious ^a^ | very serious ^b,c^ | not serious | not serious | none | 937/6361 (14.7%) | 215/1506 (14.3%) | **RR 1.25** (0.76 to 2.05) | **70 fewer per 1.000** (from 160 fewer to 20 more) | ⨁◯◯◯ VERY LOW |  |
| **BPD (any definition)** | | | | | | | | | | | | |
| 7 | observational studies | serious ^a^ | very serious ^b,c^ | not serious | not serious | strong association | 1749/6469 (27.0%) | 916/1552 (59.0%) | **RR 0.36** (0.27 to 0.47) | **360 more per 1.000** (from 260 more to 470 more) | ⨁◯◯◯ VERY LOW |  |
| **NEC (any stage)** | | | | | | | | | | | | |
| 6 | observational studies | serious ^a^ | very serious ^b,c^ | not serious | not serious | publication bias strongly suspected strong association ^d^ | 437/6022 (7.3%) | 228/1513 (15.1%) | **RR 0.49** (0.35 to 0.68) | **70 more per 1.000** (from 20 more to 120 more) | ⨁◯◯◯ VERY LOW |  |
| **IVH (any grade)** | | | | | | | | | | | | |
| 7 | observational studies | serious ^a^ | very serious ^b,c^ | not serious | not serious | none | 896/6492 (13.8%) | 382/1528 (25.0%) | **RR 0.65** (0.48 to 0.88) | **90 more per 1.000** (from 30 more to 150 more) | ⨁◯◯◯ VERY LOW |  |
| **ROP (any stage)** | | | | | | | | | | | | |
| 6 | observational studies | serious ^a^ | very serious ^b,c^ | not serious | not serious | publication bias strongly suspected strong association ^d^ | 425/5792 (7.3%) | 381/1354 (28.1%) | **RR 0.23** (0.18 to 0.31) | **211 fewer per 1.000** (from 219 fewer to 200 fewer) | ⨁◯◯◯ VERY LOW |  |

CI: Confidence interval; RR: Risk ratio

*Explanations*

a. Failure to adequately control confounding
b. Statistical test for heterogeneity shows a P value <0.05
c. The I^2^ is >50%
d. Asymmetry of funnel plots

**Supplement 5 –** Forrest plots of cohort studies including patients without PDA

a)
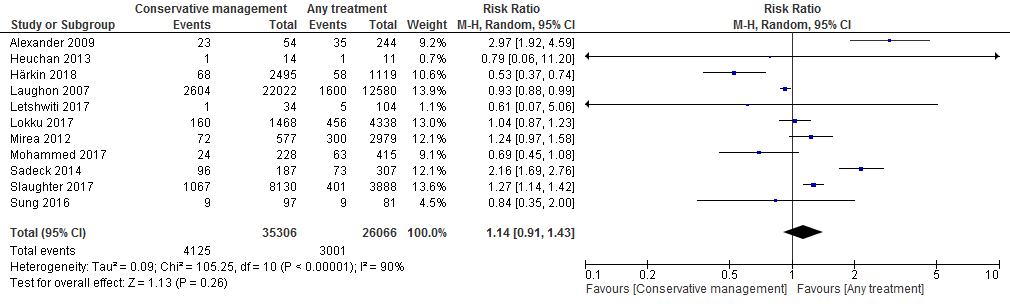


b)
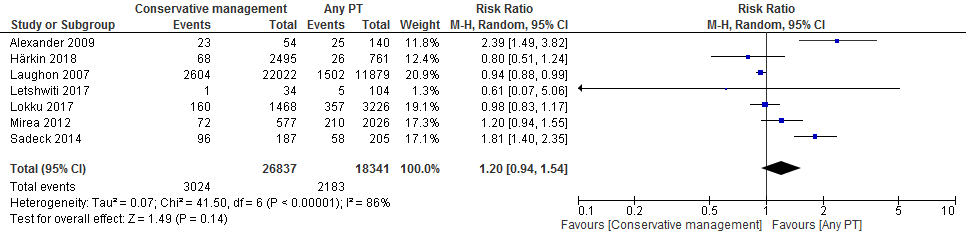


c)
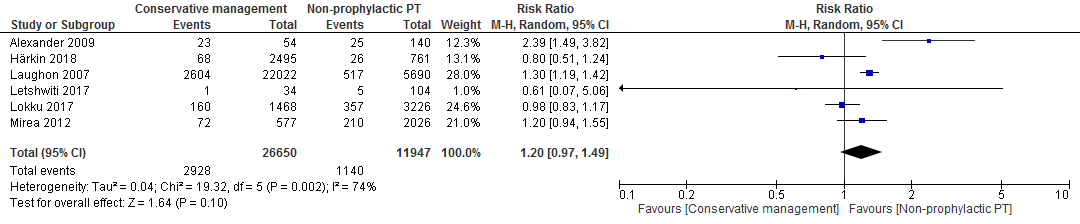


d)
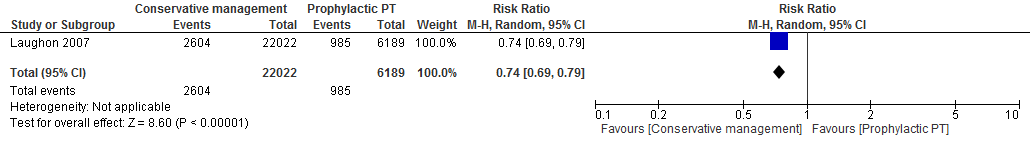


e)
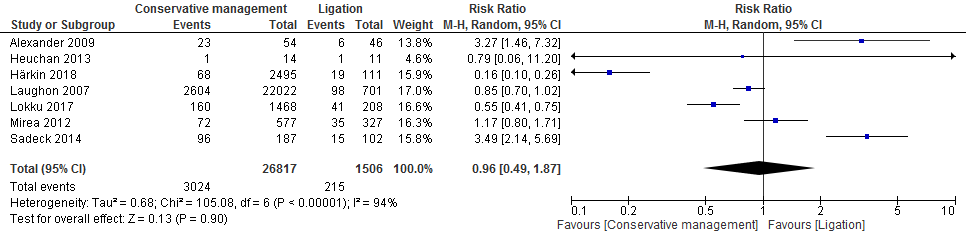


**Figure S5-1 –** Conservative management versus a) any treatment; b) any pharmacological treatment; c) non-prophylactic pharmacological treatment; d) prophylactic pharmacological treatment, and; e) ligation for patent ductus arteriosus in cohort studies including patients without patent ductus arteriosus – Mortality

a)
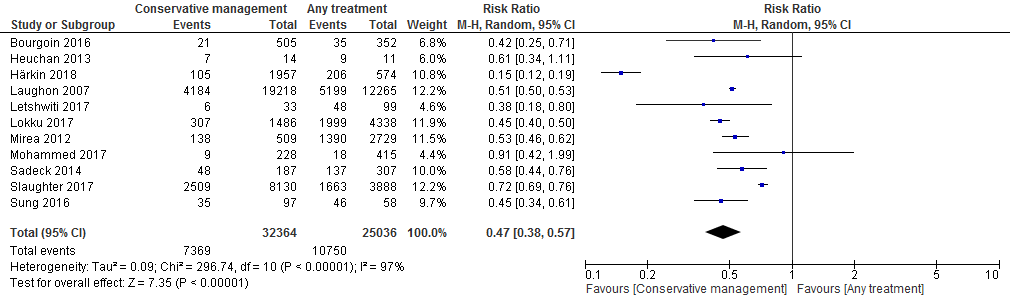


b)
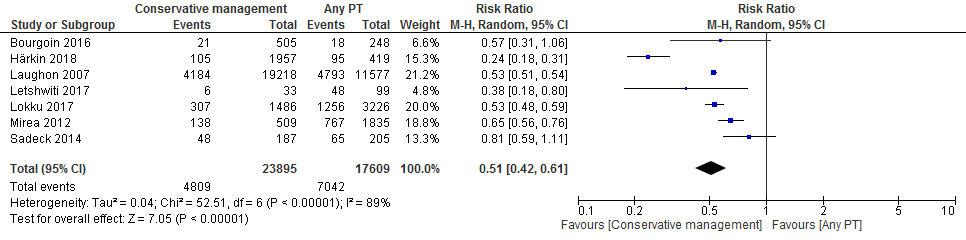


c)


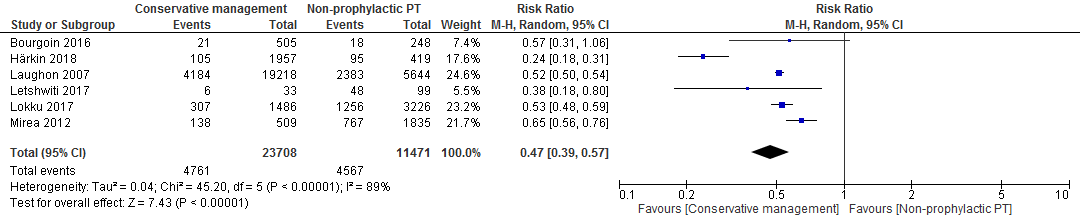


d)


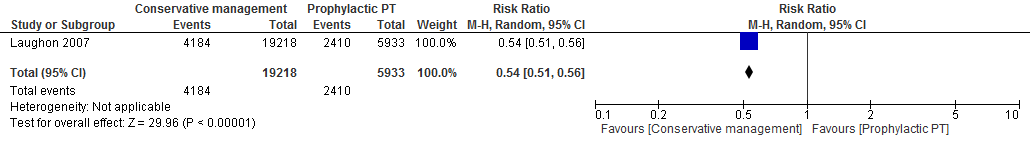


e)


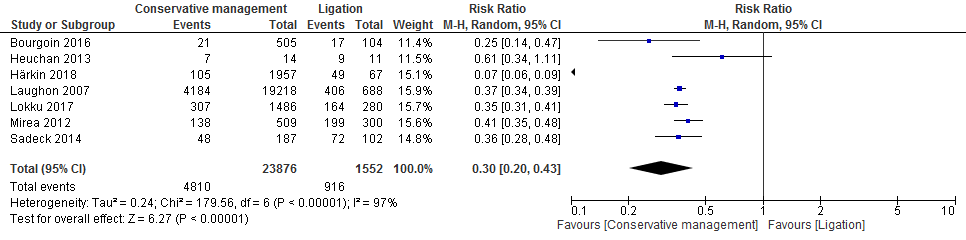


**Figure S5-2 –** Conservative management versus a) any treatment; b) any pharmacological treatment; c) non-prophylactic pharmacological treatment; d) prophylactic pharmacological treatment, and; e) ligation for patent ductus arteriosus in cohort studies including patients without patent ductus arteriosus – BPD (any definition)

a)
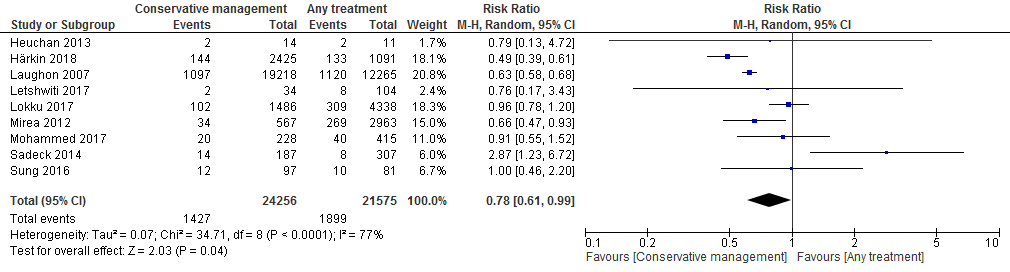


b)


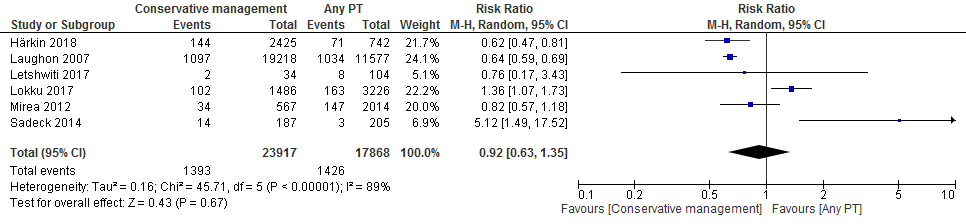


c)
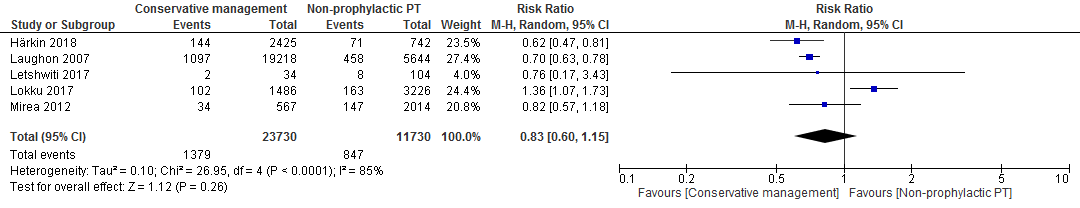


d)


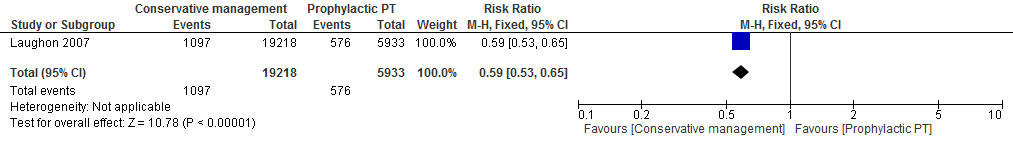


e)


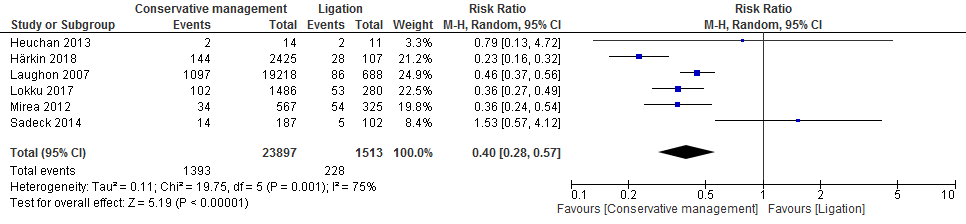


**Figure S5-3 –** Conservative management versus a) any treatment; b) any pharmacological treatment; c) non-prophylactic pharmacological treatment; d) prophylactic pharmacological treatment, and; e) ligation for patent ductus arteriosus in cohort studies including patients without patent ductus arteriosus - NEC (any stage)

a)


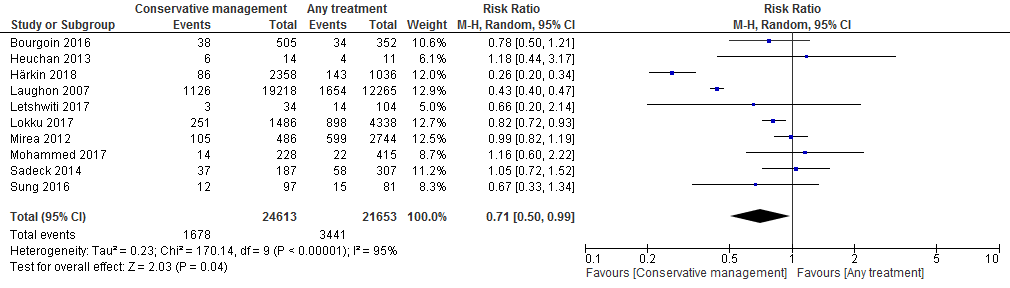


b)
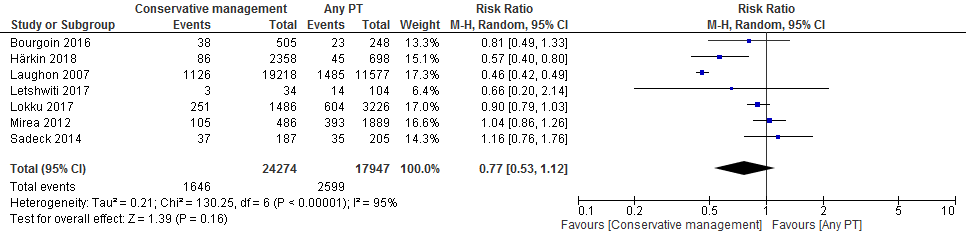


c)


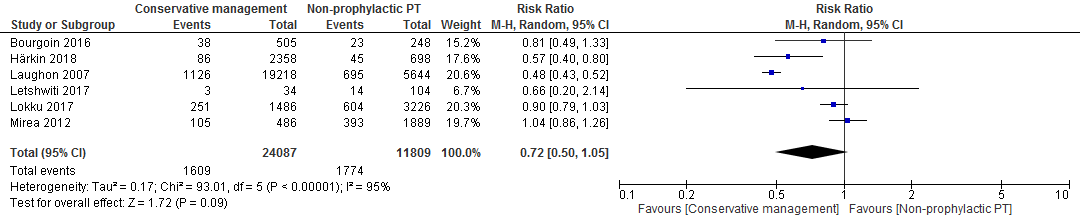


d)


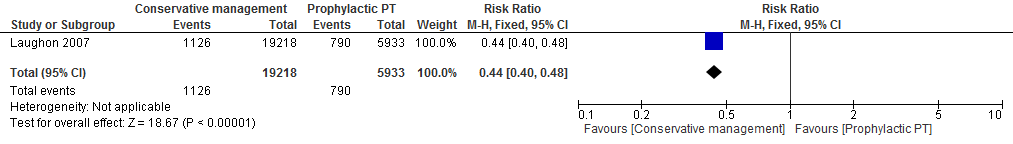


e)


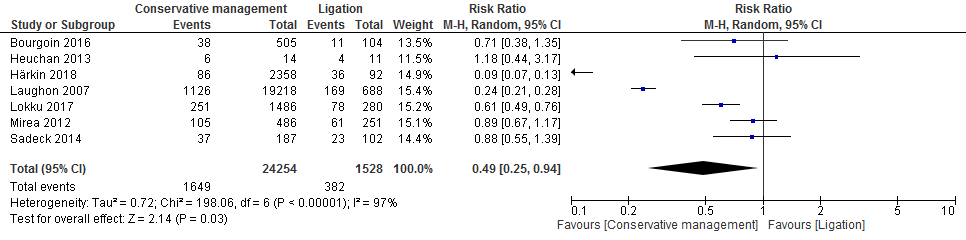


**Figure S5-4 –** Conservative management versus a) any treatment; b) any pharmacological treatment; c) non-prophylactic pharmacological treatment; d) prophylactic pharmacological treatment, and; e) ligation for patent ductus arteriosus in cohort studies including patients without patent ductus arteriosus – IVH (any stage)

a)


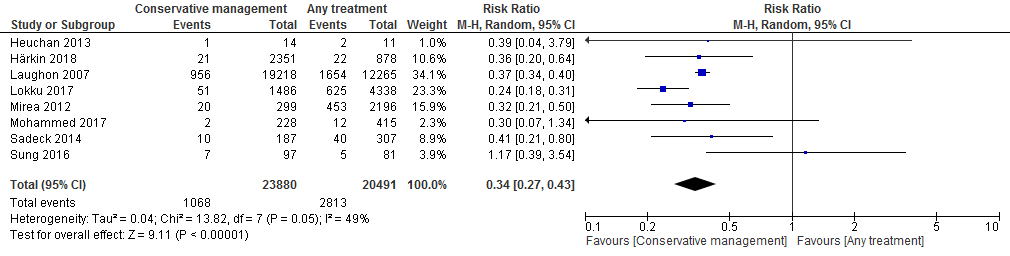
b)


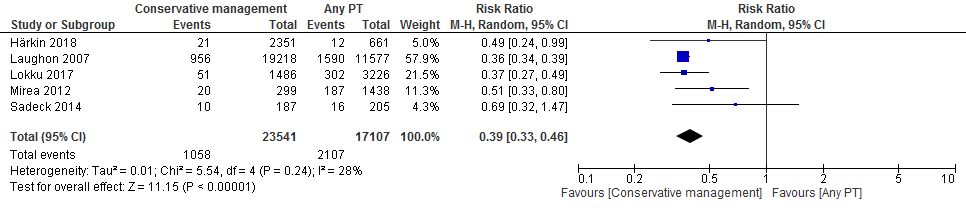


c)


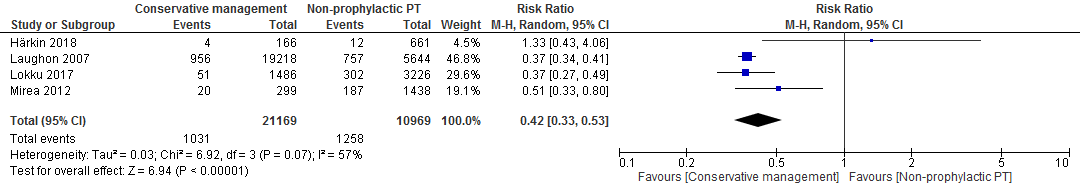


d)


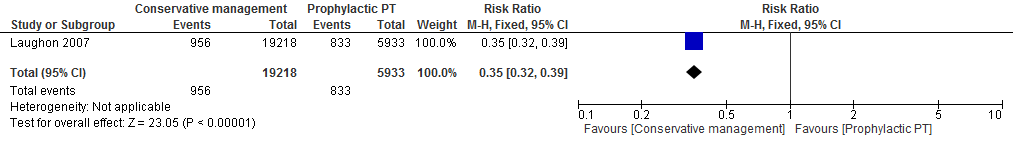


e)


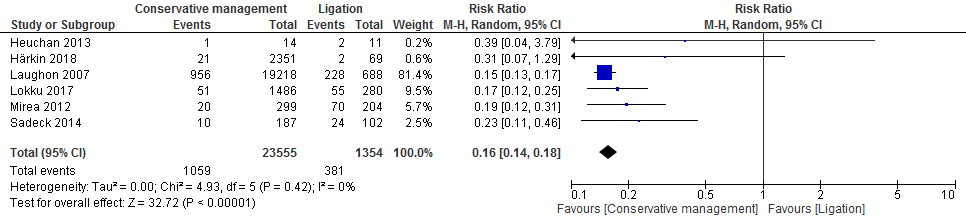


**Figure S5-5 –** Conservative management versus a) any treatment; b) any pharmacological treatment; c) non-prophylactic pharmacological treatment; d) prophylactic pharmacological treatment, and; e) ligation for patent ductus arteriosus in cohort studies including patients without patent ductus arteriosus – ROP (any stage)
